# Effects of g-C_3_N_4_ Heterogenization into Intrinsically Microporous Polymers on the Photocatalytic Generation of Hydrogen Peroxide

**DOI:** 10.1021/acsami.1c23960

**Published:** 2022-04-24

**Authors:** Yuanzhu Zhao, Lina Wang, Richard Malpass-Evans, Neil B. McKeown, Mariolino Carta, John P. Lowe, Catherine L. Lyall, Rémi Castaing, Philip J. Fletcher, Gabriele Kociok-Köhn, Jannis Wenk, Zhenyu Guo, Frank Marken

**Affiliations:** † Department of Chemistry, University of Bath, Claverton Down, Bath BA2 7AY, UK; ‡ EaStCHEM School of Chemistry, University of Edinburgh, Joseph Black Building, David Brewster Road, Edinburgh, Scotland EH9 3JF, UK; § Department of Chemistry, Swansea University, College of Science, Grove Building, Singleton Park, Swansea SA2 8PP, UK; ∥ University of Bath, Materials & Chemical Characterisation Facility, MC^2^, Bath BA2 7AY, UK; ⊥ Department of Chemical Engineering and Water Innovation Research Centre, WIRC, University of Bath, Claverton Down, Bath BA2 7AY, UK; # Department of Chemical Engineering, Imperial College London, South Kensington Campus, London, SW7 2AZ, UK

**Keywords:** disinfection, hydrogen generation, adsorption, bipolar photocatalysis, hydrogen peroxide

## Abstract

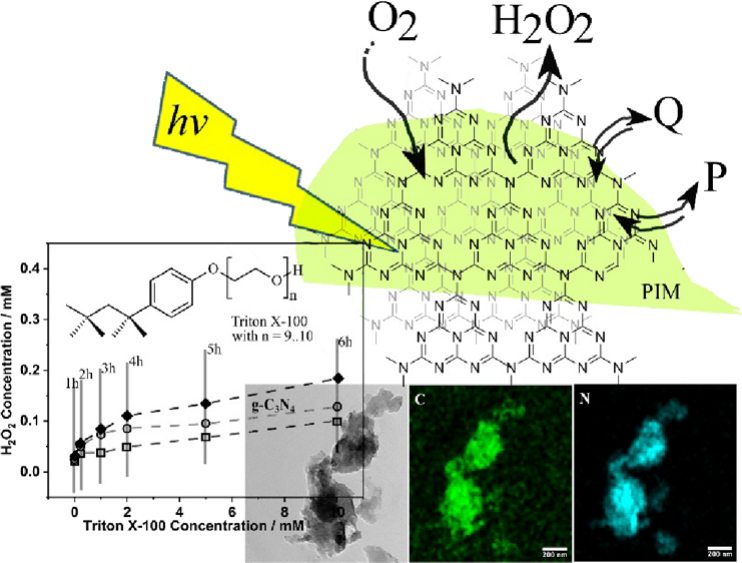

Graphitic carbon nitride (g-C_3_N_4_) is known to photogenerate hydrogen peroxide in the presence of hole quenchers in aqueous environments. Here, the g-C_3_N_4_ photocatalyst is embedded into a host polymer of intrinsic microporosity (PIM-1) to provide recoverable heterogenized photocatalysts without loss of activity. Different types of g-C_3_N_4_ (including Pt@g-C_3_N_4_, Pd@g-C_3_N_4_, and Au@g-C_3_N_4_) and different quenchers are investigated. Exploratory experiments yield data that suggest binding of the quencher either (i) directly by adsorption onto the g-C_3_N_4_ (as shown for α-glucose) or (ii) indirectly by absorption into the microporous polymer host environment (as shown for Triton X-100) enhances the overall photochemical H_2_O_2_ production process. The amphiphilic molecule Triton X-100 is shown to interact only weakly with g-C_3_N_4_ but strongly with PIM-1, resulting in accumulation and enhanced H_2_O_2_ production due to the microporous polymer host.

## 1 Introduction

Hydrogen peroxide is a crucial chemical reagent in many fields of application including green epoxidation chemistry,^1^ pollutant treatment,^2^ surface cleaning,^3^ solar disinfection,^4^ bleaching of pulp,^5^ health and wound cleaning,^6^ or for electrochemical and colorimetric biosensor applications.^7^ Hydrogen peroxide is employed in nature/biological systems, for example, during inflammation^8^ and in peroxisome processes.^9^ The production of hydrogen peroxide is possible from molecular oxygen by chemical reduction, for example, the BASF anthraquinone process based on anthraquinol and air^10,11^ or by direct electrochemical reduction on carbon electrodes.^10,12^ Direct reaction of hydrogen and oxygen gas has been demonstrated over heterogeneous catalysts to yield up to 56 mM H_2_O_2_ in aqueous media.^13^ Many sacrificial reducing agents (or pollutants) react in the presence of catalyst with molecular oxygen to give hydrogen peroxide.^14^ In nature, peroxidases^15^ (e.g., glucose peroxidase^16^) are able to generate H_2_O_2_ and/or to use H_2_O_2_ in oxidation reactions. Reports have emerged on the photochemical production of hydrogen peroxide directly from water and O_2_.^17^ However, thermodynamically, hydrogen peroxide is unstable and likely to dismutate back into H_2_O and one-half O_2_.^18^

**Figure 1:**
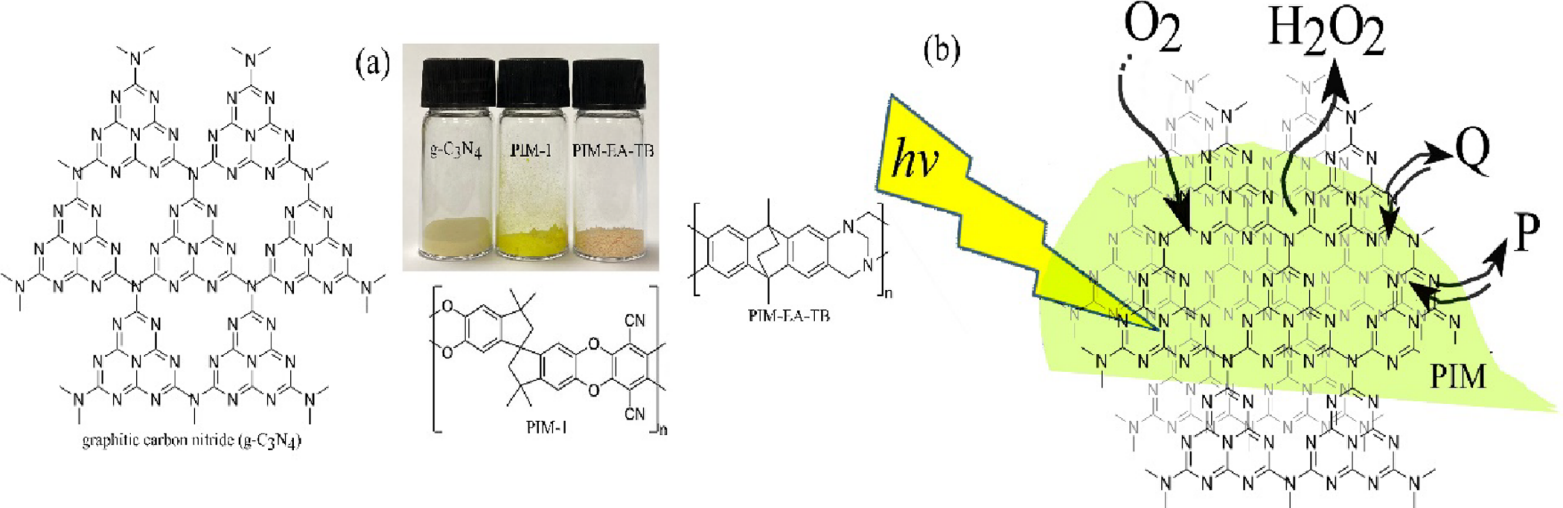
(a) Photograph and molecular structures of g-C_3_N_4_ (tentative), PIM-1, and PIM-EA-TB. (b) Illustration of the mechanism for photochemical hydrogen peroxide production.

Photocatalytic production of H_2_O_2_^19,20^ is commonly observed when oxygen is allowed to interact with the photocatalyst in the presence of hole quencher materials (e.g., alcohols,^21^ oxalic acid,^22^ or other organic donors^23^). Hydrogen peroxide generation is possible with graphitic carbon nitride photocatalysts (g-C_3_N_4_; see Figure 1a; note that only the intermediate heptazine structure is shown as illustration, although further condensation into more defective structures at higher temperatures is likely;^24^ CAS no. 290-87-9), which was synthesized as early as 1834 by Berzelius.^25^ g-C_3_N_4_ has been developed and used in more recent work by Antonietti and co-workers^26,27^ in 2006 and by Wang et al.^28^ in 2009 for applications in wastewater treatment^29,30^ and in photochemical hydrogen production.^31,32^ Graphitic carbon nitride g-C_3_N_4_ has a layered structure and a typical bandgap of 2.7 eV up to 5.0 eV depending on structural variations and modifications.^26^ The g-C_3_N_4_ surface charge is characterized by a point of zero charge (pzc) at pH 4.2.^33^ Conventional graphitic carbon nitride adsorbs light at λ = 420 nm and therefore exhibits a pale yellow coloration (see Figure 1). Many derivatives of g-C_3_N_4_ have been developed to improve photocatalysis performance,^34^ and recent reviews^35,36^ provide a good introduction to this versatile organic photocatalytic material. Particulate g-C_3_N_4_ can be employed as suspended particles^37^ or 2D nanoparticles,^38^ coated onto surfaces,^39^ associated with other photocatalysts,^40^ or embedded into polymers^41^ or porous host materials.^42,43^ It has been reported that g-C_3_N_4_ in conjunction with graphene can be employed to photogenerate H_2_O_2_.^44^ Defect engineering has been employed to increase rates of H_2_O_2_ production.^45^

Polymers of intrinsic microporosity (PIMs) are molecularly stiff materials composed of contorted ladder-like structures^46^ (e.g., the most-studied PIM-1 and PIM-EA-TB in Figure 1). This leads to good solvent processability (due to molecular interactions in the solid being weak^47^), and uniformly microporous film deposits cast from solution with typically 1 nm diameter pores.^48^ Applications for PIMs have emerged in gas permeation and separation^49^ as well as in liquid phase systems such as electrochemical analysis,^50^ electroosmotic processes,^51^ or electrochemical energy storage.^52^ Both PIM-1 and PIM-EA-TB have been employed previously for embedding catalysts^53^ with the aim of minimizing catalyst surface blocking by avoiding detrimental PIM-catalyst interactions (due to molecular rigidity in the polymer backbone) and maximizing catalyst performance (due to a fully accessible catalyst surface^54^). We have recently demonstrated photocatalytic hydrogen production with a co-catalyst-modified g-C_3_N_4_ embedded into a PIM.^55^

Here, we investigate g-C_3_N_4_ photocatalysts for H_2_O_2_ generation (i) suspended in aqueous solution, (ii) coated with a PIM material and suspended as particles, or (iii) heterogenized when embedded into PIM-1 or PIM-EA-TB and deposited onto a filter paper substrate. In this study, the heterogenization of g-C_3_N_4_ photocatalysts into polymers of intrinsic microporosity is demonstrated to give highly active films (recoverable from solution) with reactivity similar to that of suspension systems. Filter paper is employed as a simple substrate for photocatalyst–polymer composites to form uniform, stable, and recoverable/reusable films. The important role of hole quencher adsorption (both directly onto g-C_3_N_4_ and indirectly into PIM-1 micropores) in the photocatalytic reaction is highlighted. Glucose is employed as quencher of choice due to its prevalence in digested biomass, for example, from cellulose. In the presence of amphiphilic molecules such as Triton X-100, PIM-1 is shown to bind the quencher and, in this way, introduce a localized high-concentration environment for enhancing photoreaction and H_2_O_2_ production.

## 2 Experimental Section

### 2.1 Reagents

Melamine, glucose, sodium oxalate, potassium hexachloroplatinate(IV), palladium(II) chloride, and potassium gold(III) chloride were purchased from Sigma-Aldrich and used without further purification. Sodium acetate trihydrate was purchased from BDH Chemicals Ltd. Triton X-100 (C_14_H_22_O(C_2_H_4_O)_10_) was obtained from Biomol GmbH. PIM-1,^56^ PIM-EA-TB,^57^ and g-C_3_N_4_^58^ were prepared following literature recipes. Ultrapure (18.2 MΩ cm at 18 °C) water from a Thermo Fisher water purification system was used for all solutions.

### 2.2 Instrumentation

Transmission electron microscopy (TEM) was performed on a JEOL JEM-2100 Plus instrument with a 200 kV maximum accelerating voltage. Energy dispersive X-ray analysis (EDX) data was collected using an Oxford Instruments X-Max^N^TSR silicon drift detector. Scanning electron microscopy (SEM) images were captured with a JEOL JSM-7900F FESEM instrument at an accelerating voltage of 5 kV. Powder X-ray diffraction (PXRD) patterns were recorded in transmission mode on a STOE STADI P equipped with a Multi-Mythen detector using monochromated Cu K_α_ radiation (1.54060 Å). Raman spectroscopy was performed at wavelengths of 325, 532, and 785 nm excitation with a Renishaw inVia confocal Raman microscope. Mass spectrometry analysis was carried out with an Automated Agilent QTOF (Walkup) used with HPLC (four chromatography columns) and a variable wavelength detector (VWD). Nitrogen gas adsorption analysis (Brunauer–Emmett–Teller or BET) for g-C_3_N_4_ and PIM-1 powder was performed with an Autosorb-iQ-C instrument by Quantachrome. NMR spectra were acquired on a 400 MHz Bruker Neo spectrometer equipped with an iProbe. Spectra were acquired unlocked in H_2_O at 298 K, and an automated shimming routine was carried out on the ^1^H signal. X-ray photoelectron spectroscopy (XPS) was performed with a Thermo Fisher K-Alpha+ facility using a monochromated microfocused Al K_α_-generated X-ray beam. The spectra were collected under ultrahigh vacuum conditions (residual pressure = 8 × 10^–8^ Pa) at a pass energy of 20 eV with a spot size of 200 μm. All binding energies were corrected to 284.8 eV (C1s). The fitting of fine scans on elements was carried out using Avantage software. A Shimadzu UV-2600 spectrophotometer was used to measure the UV–visible diffuse-reflectance spectroscopy (DRS) using BaSO_4_ as a substrate. The light source in photochemical experiments was a Thorlabs M385LP1 with nominal 1200 mW 385 nm light. The intensity is nominal at 0.23 mW cm^–2^ in a 20 cm distance. A power meter (Gentec Electro-Optics, Inc. Canada) was employed to confirm the light intensity at the distance of 2 cm from the light source as 80 mW cm^–2^.

### 2.3 Procedures

#### 2.3.1 Synthesis of the g-C_3_N_4_ Materials

Graphitic carbon nitride was obtained by heating melamine at 550 °C in a tube furnace for 4 h in a crucible with lid in ambient air. The yellow product was ground in a mortar to give a uniform product (typically 30% product yield by weight). The yellow powder was further modified by photochemical metal deposition following a literature recipe.^59^ Typically, 0.4 g of g-C_3_N_4_ and 0.04 g of metal precursor salt (K_2_PtCl_6_/ PdCl_2_/ KAuCl_4_) were mixed in 20 mL of saturated sodium oxalate solution (with a pH of approx. 8), forming a suspension. After suspending solids aided by an ultrasonic cleaning bath for 15 min, the suspension was stirred with a closed lid and illuminated with a 385 nm light from a blue LED (2 cm distance, approx. 80 mW cm^–2^) for 72 h. The product appeared dark gray in coloration and was filtered, washed with water, and dried.

#### 2.3.2 Embedding Photocatalysts into Films

To immobilize g-C_3_N_4_@PIM-1 composite onto a filter paper, g-C_3_N_4_ and PIM-1 with a 5:1 weight ratio were added into chloroform (5 mg g-C_3_N_4_ and 1 mg PIM-1 in 1 cm^3^) and suspended by ultrasonication for 15 min. The composite was drop-cast deposited onto filter paper (Whatman, pore size less than 2 μm, cut into a size of 4 cm × 1 cm strips). After drying in air, the composite-immobilized filter paper was immersed in aqueous solution and employed in photochemical reactions.

#### 2.3.3 PIM-1 Particles and g-C_3_N_4_@PIM-1 Particles

**Figure 2:**
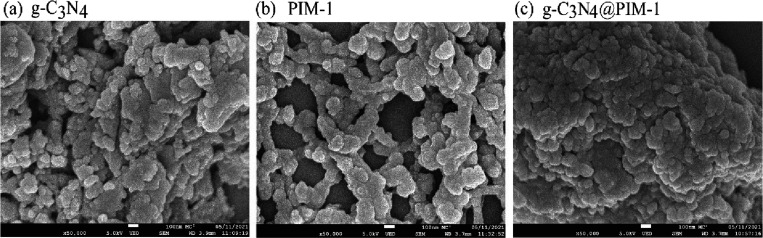
SEM images of (a) g-C_3_N_4_ particles, (b) PIM-1 particles, and (c) particulate composite of g-C_3_N_4_@PIM-1 particles with weight ratio of 5:1 g-C_3_N_4_:PIM-1.

PIM-1 nanoparticles were synthesized with an anti-solvent precipitation method according to a literature method with a slight modification.^60^ Typically, 3 mL of PIM-1 solution in chloroform (with a concentration of approx. 15 mg mL^–1^) was added dropwise into 20 mL of methanol with vigorous stirring. The stirring was continued for 4 h. Then, the obtained suspension was centrifuged at 5000 rpm for 30 min. Excess methanol was removed, and the solid phase was dried in an oven at 80 °C overnight. SEM images reveal aggregated particles with typically 100–200 nm diameter (Figure 2b). Particles of g-C_3_N_4_@PIM-1 were prepared by anti-solvent precipitation in 20 mL of methanol using g-C_3_N_4_ and PIM-1 in a weight ratio of 5:1 in chloroform. An SEM image in Figure 2c shows aggregated g-C_3_N_4_ with PIM-1. Surface analysis by nitrogen gas absorption (BET; see Supporting Information) suggests for g-C_3_N_4_ a surface area of 36.4 m^2^ g^–1^ and for PIM-1 a surface area of 875 m^2^ g^–1^. Therefore, in composites, PIM-1 is likely to dominate in terms of adsorption behavior.

#### 2.3.4 Photochemical Reactions

A glass vial with 20 mL of solution was charged either with g-C_3_N_4_ powder (5 mg) or with g-C_3_N_4_-modified filter paper (5 mg of g-C_3_N_4_ with 1 mg of PIM deposited onto a 1 cm × 4 cm area). Photochemical reactions were performed at ambient temperature and pressure (20% oxygen) unless stated otherwise. Magnetic stirring was applied when exposed to LED light (Thorlabs, M385LP1 with 1200 mW, 385 nm light in an approx. 2 cm distance; intensity of approx. 80 mW cm^–2^). For Ar/O_2_ control experiments, the photochemical solution was purged with Ar/O_2_ for 30 min prior to irradiation. During the photoelectrochemical experiment, a continuous gas flow (Ar/O_2_) was maintained.

#### 2.3.5 Detection of Hydrogen Peroxide

Quantitative analysis of the hydrogen peroxide concentration was performed following a literature method.^61^ Briefly, H_2_O_2_ was reacted with *para*-nitrophenyl boronic acid to give *para*-nitrophenol, which was quantified by mass spectrometry coupled to HPLC (Automated Agilent QTOF; see details in the Supporting Information).

#### 2.3.6 Quantitative Concentration Analysis by NMR

^1^H NMR spectra were obtained in H_2_O with single-solvent suppression using presaturation (Bruker pulse program noesygppr1d) to suppress the water signal. The relaxation delay was set to 30 s to allow for the accurate integration of peaks. A small amount of dimethyl sulfoxide was added to sample solutions as an internal ^1^H-NMR calibration standard. The detailed experimental process is reported in the Supporting Information. For the glucose binding experiment, peaks at 5.01 and 3.01 ppm are selected for α-d-glucose and β-d-glucose concentration analyses, respectively (with the DMSO peak at 2.50 ppm; see Figure S2 in the Supporting Information). For Triton X-100 binding experiments, the peak at 7.02 ppm (two aromatic protons) is selected with the DMSO peak at 2.50 ppm (see Figure S3 in the Supporting Information).

## 3 Results and Discussion

### 3.1 Photogeneration of Hydrogen Peroxide I: Effect of PIM Host Materials

Initial experiments were performed with glucose as the quencher for photogenerated holes in g-C_3_N_4_. The g-C_3_N_4_ material employed here has been reported previously^59^ and is based on a disordered layered structure probably containing heptazine units or more condensed and defective layers.^62^ A detailed identification of structural motifs is difficult but has been suggested as an example based on ^13^C-MAS-NMR methods.^63^ Here, X-ray diffraction data in Figure S5 confirm the main diffraction peaks for the 100 and 002 planes.^64^ Transmission electron microscopy (Figure S6) and electron diffraction are consistent with X-ray diffraction. Raman data in Figure S7 were obtained with 325 nm excitation (data obtained with 532 and 785 nm excitation suffer from strong fluorescent backgrounds). The main Raman bands are consistent with literature reports for g-C_3_N_4_.^65^ Diffuse-reflectance UV/Vis data (Supporting Information, Figure S8) and XPS data (Supporting Information, Figure S9) are consistent with the literature reports.^28,58^

**Figure 3:**
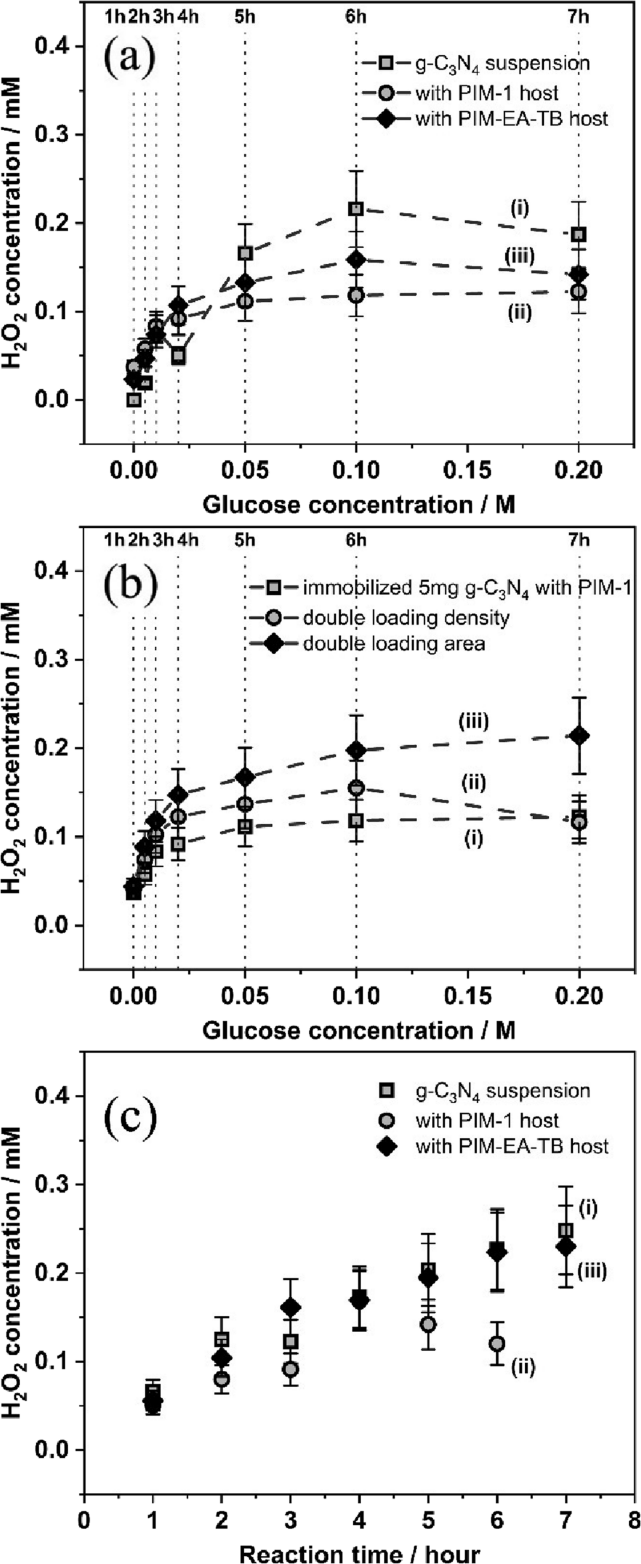
(a) Photogeneration of H_2_O_2_ with (i) 5 mg of g-C_3_N_4_ in suspension, (ii) 6 mg of g-C_3_N_4_@PIM-1 (containing 5 mg ofg-C_3_N_4_) immobilized onto 4 cm × 1 cm filter paper, and (iii) 6 mg of g-C_3_N_4_@PIM-EA-TB (containing 5 mg of g-C_3_N_4_) immobilized onto 4 cm × 1 cm filter paper (in 20 mL of solution with stepwise addition of glucose; 385 nm LED). (b) As above, (i) 6 mg of g-C_3_N_4_@PIM-1 on 4 cm × 1 cm filter paper, (ii) 12 mg of g-C_3_N_4_@PIM-1 on 4 cm × 1 cm filter paper, and (iii) 12 mg of g-C_3_N_4_@PIM-1 on 4 cm × 2 cm filter paper. (c) Plot of H_2_O_2_ concentration versus time with (i) 5 mg of g-C_3_N_4_ in suspension, (ii) 6 mg of g-C_3_N_4_@PIM-1 (containing 5 mg of g-C_3_N_4_) immobilized onto 4 cm × 1 cm filter paper, and (iii) 6 mg of g-C_3_N_4_@PIM-EA-TB (containing 5 mg of g-C_3_N_4_) immobilized onto 4 cm × 1 cm filter paper immersed in 20 mL of 0.1 M glucose solution. Estimated errors in all data points are ±20%.

Figure 3a shows data for the production of H_2_O_2_ with time and with increasing glucose concentration. With 5 mg of g-C_3_N_4_ suspended in 20 mL of solution and with 100 mM glucose in solution under constant stirring and illumination (LED, λ = 385 nm), a maximum of 216 μM H_2_O_2_ is observed after 6 h of reaction. A higher glucose concentration or a longer reaction time did not increase the yield. Next, the experiment was repeated but with 5 mg of g-C_3_N_4_ immobilized onto a filter paper (area, 4 cm^2^) either with PIM-1 or with PIM-EA-TB (1 mg of PIM together with 5 mg of g-C_3_N_4_). Data in Figure 3a suggest very similar trends and, although the photocatalyst is immobilized, up to approx. 100 μM H_2_O_2_ were obtained after 6 h in 100 mM glucose solution. Therefore, the photocatalyst remains active when embedded into either microporous PIM-1 or microporous PIM-EA-TB with access to both dissolved oxygen and glucose diffusing through the microporous hosts. The reaction (simplified) can be expressed tentatively/schematically as in eqs 1–3.
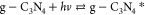
1
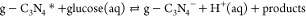
2
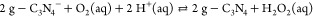
3

For the photochemical process to be effective, both glucose(aq) and O_2_(aq) have to interact closely with the g-C_3_N_4_ surface. Both reagents also have to permeate through the PIM host materials. To explore the effects of the film thickness and catalyst loading, further glucose addition experiments were performed. In Figure 3b, data are shown comparing (i) 5 mg of g-C_3_N_4_ in PIM-1 over 4 cm^2^ with (ii) 10 mg of g-C_3_N_4_ in PIM-1 over 4 cm^2^ and with (iii) 10 mg of g-C_3_N_4_ in PIM-1 over 8 cm^2^. Only when using an area of 8 cm^2^ is the H_2_O_2_ production doubled, and therefore, the active geometric area is important. This suggests that for thicker g-C_3_N_4_@PIM-1 film deposits, not all the photocatalyst in the film is fully active (potentially due to limited light penetration or due to transport limitations with O_2_ or glucose not reaching all of the catalyst surface in the immobilized film).

Figure 3c shows H_2_O_2_ production data for 100 mM glucose solution and as a function of time for (i) 5 mg of g-C_3_N_4_ suspension, (ii) 5 mg of g-C_3_N_4_ in PIM-1, and (iii) 5 mg of g-C_3_N_4_ in PIM-EA-TB. All three systems allow H_2_O_2_ production, but the catalyst in PIM-1 appears to lose some activity after 6 h of continuous photocatalytic reaction. It was recently reported that PIM-1 is itself photochemically active and that some photodegradation of PIM-1 is possible and probably the cause for detrimental changes in porosity and transport.^66,67^ However, PIM-EA-TB appears to exhibit a more stable reactivity (consistent with that of an equivalent amount of photocatalyst in a stirred suspension) under these conditions. More extensive long-term photocatalyst stability testing is under investigation and will be reported separately.

The production of hydrogen peroxide is observed without/with the presence of PIM-1 or PIM-EA-TB, and a plateauing of reactivity with increased glucose concentration occurs in all cases. This has recently been suggested to be linked to binding (assumed Langmuirian)^43^ of the hole quencher (here glucose) onto the g-C_3_N_4_ surface (vide infra). A further factor in the plateauing of H_2_O_2_ production can be the decomposition of H_2_O_2_ (competing with H_2_O_2_ production) either in solution or under conditions of photocatalysis in the catalyst film. Further data for H_2_O_2_ production are summarized in Table 1. With 5 mg of g-C_3_N_4_ suspended and without glucose quencher, no significant production of H_2_O_2_ occurs. However, for g-C_3_N_4_ in PIM-1 even without glucose, some H_2_O_2_ is produced. Therefore, it seems likely that some degradation of the PIM-1 host polymer may occur under these conditions.

**Table 1:** Comparison of g-C_3_N_4_ Photocatalyst Performance for Photosynthesis of Hydrogen Peroxide^a^

catalyst (5 mg g-C_3_N_4_)	reaction method	quencher	reaction condition	H_2_O_2_ concentration
g-C_3_N_4_	suspension	H_2_O	ambient	none
g-C_3_N_4_	suspension	0.1 M glucose	ambient	66 ± 13 μM
g-C_3_N_4_ (10 mg g-C_3_N_4_)	suspension	0.1 M glucose	ambient	130 ± 26 μM
g-C_3_N_4_	suspension	0.1 M acetate	ambient	30 ± 6 μM
g-C_3_N_4_ with PIM-1	immobilized on filter paper	H_2_O	ambient	37 ± 7 μM
g-C_3_N_4_ with PIM-1	immobilized on filter paper	0.1 M glucose	ambient	51 ± 10 μM
g-C_3_N_4_ with PIM-1	immobilized on filter paper	0.1 M acetate	ambient	41 ± 8 μM
g-C_3_N_4_ with PIM-1	immobilized on filter paper	0.1 M glucose	under Ar flow	none
g-C_3_N_4_ with PIM-1	immobilized on filter paper	0.1 M glucose	under Ar flow	39 ± 8 μM
g-C_3_N_4_ with PIM-1	immobilized on filter paper	0.1 M glucose	under O_2_ flow	77 ± 15 μM

a λ = 385 nm, 80 mW cm^-2^, reaction time: 1 h, stirred solution. Errors estimated are ±20%.

In the presence of 100 mM glucose, typically 66 μM H_2_O_2_ is detected within the suspension after 1 h of photocatalysis. Doubling the amount of photocatalyst doubles the H_2_O_2_ yield. When employing g-C_3_N_4_@PIM-1 immobilization on the filter paper substrate, 51 μM H_2_O_2_ is produced with same concentration of glucose, which is very similar to the yield for suspended g-C_3_N_4_. When using 100 mM sodium acetate as the quencher, both g-C_3_N_4_ suspension and g-C_3_N_4_@PIM-1 immobilization on the filter paper produce similar amounts of H_2_O_2_ (but lower compared to those produced with glucose). Clearly, each type of quencher produces specific effects that are linked to either the transport in the microporous environment and/or the interaction of the quencher with the photocatalyst.

Control experiments under Ar/ O_2_ flow were performed to explore the role of oxygen during photochemical reactions. When 5 mg of photocatalyst was immobilized on the filter paper with 1 mg of PIM-1, no hydrogen peroxide was detected after 1 h of photocatalysis in the argon-saturated glucose solution. With the same concentration of glucose in solution and saturated with pure O_2_ prior to irradiation, g-C_3_N_4_@PIM-1 immobilized on filter paper generates an increased amount of H_2_O_2_ (77 ± 15 μM) when compared with that generated in ambient air (39 ± 8 μM). It can be concluded that the presence of oxygen played a crucial role in the photochemical reactions to form hydrogen peroxide.

### 3.2 Photogeneration of Hydrogen Peroxide II: Effect of Glucose Adsorption onto g-C_3_N_4_

**Figure 4:**
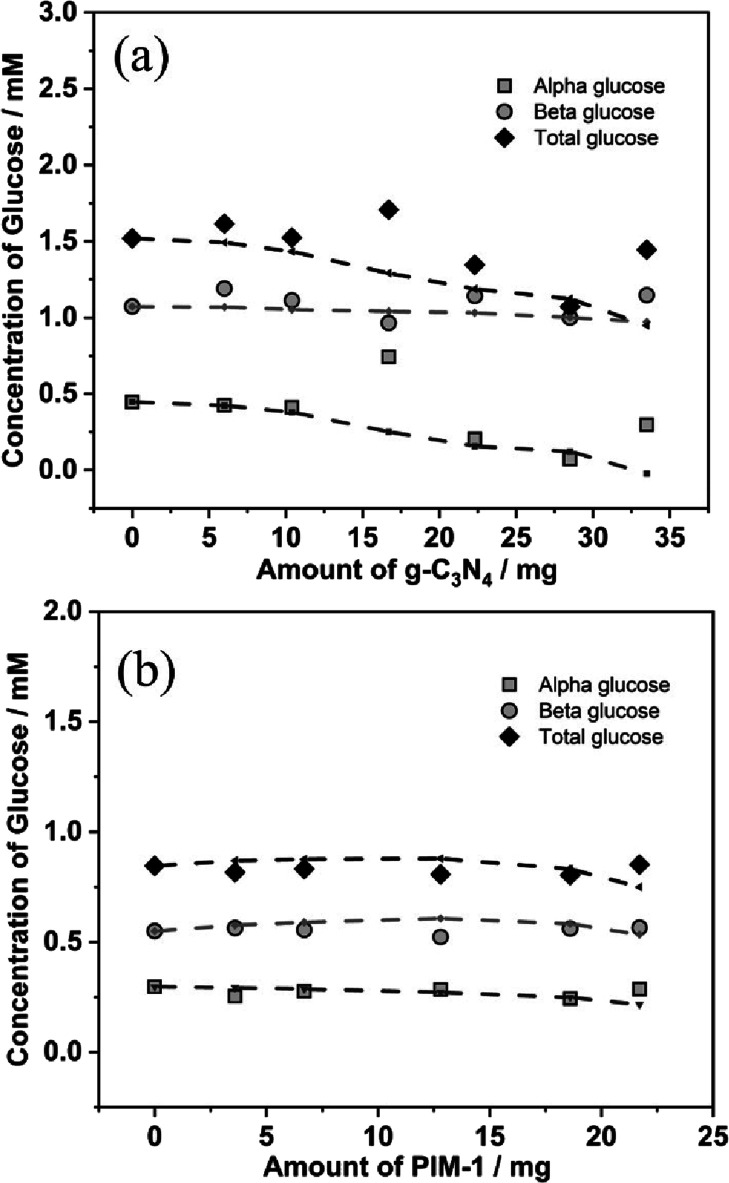
(a) Plot of glucose concentration (α-, β-, and total glucose) versus added g-C_3_N_4_ powder (determined by ^1^H-NMR). Lines correspond to best fit trends based on the competitive Langmuirian binding of α- and β-glucose with *K*_α-glucose_ = 200 (± 50) mol^–1^ dm^3^ and *K*_β-glucose_ < 10 (± 5) mol^–1^ dm^3^. (b) As above, but for the addition of PIM-1 nanoparticles. No significant binding of glucose to PIM-1 is observed. Estimated error in all data points is ±20%.

To better understand the photocatalytic mechanism in the presence of glucose, a binding assay for glucose onto g-C_3_N_4_ was performed with the help of ^1^H-NMR tools. A solution of glucose in water (H_2_O) was spiked with a small amount of DMSO (as an internal ^1^H-NMR standard). The concentration of glucose in H_2_O was then determined (employing water signal suppression pulses) as a function of added g-C_3_N_4_ or added PIM-1 particles. Figure 4a shows data for the concentration changes for both α-glucose (approx. 30%) and β-glucose (approx. 70%) as a function of added g-C_3_N_4_. A significant change in α-glucose concentration is observed with a theory line added based on (i) the BET surface area, (ii) an assumed binding area of 12.7 × 10^–2^ m^2^, and (iii) the assumption of a simple Langmuirian binding constant (estimated based on a competitive binding model for α- and β-glucose competing for the same binding sites) of approx. *K*_α-glucose_ = 200 (± 50) mol^–1^ dm^3^.

The effect on the β-glucose concentration was much less obvious, and no binding constant was obtained. The preferred adsorption of α-glucose onto g-C_3_N_4_ is inconsistent with the reported binding preference of β-glucose (the more polar and therefore dominant species in water) toward boronic acid-modified surfaces^68^ or toward mineral surfaces.^69^ This behavior may be linked to specific interactions of α/β-glucose to the g-C_3_N_4_ surface. The binding constant *K*_α-glucose_ suggests α-glucose half-coverage at 5 mM α-glucose (or, based on a theoretical equilibrium content of 36% α-glucose, this suggests a total glucose concentration of 14 mM for half-coverage). This fits very well with the observed onset of photoactivity in the glucose concentration range of 1 to 10 mM.

Similarly, it is possible to investigate the interaction of glucose with the PIM-1 host material (added as particles to give a PIM-1 suspension). Figure 4b shows data for the binding of glucose into PIM-1. Both α-glucose and β-glucose show only weak/insignificant interaction and no quantifiable binding isotherm. Therefore, for glucose photocatalysis, the direct interaction of α-glucose with the g-C_3_N_4_ photocatalyst appears to be essential for effective hole quenching and H_2_O_2_ production. Further surface binding effects to the photocatalyst may also affect the formation/decay of reaction intermediates/products (which are currently unknown) from glucose photodegradation.

### 3.3 Photogeneration of Hydrogen Peroxide III: The Effect of Photocatalyst Modification

**Figure 5:**
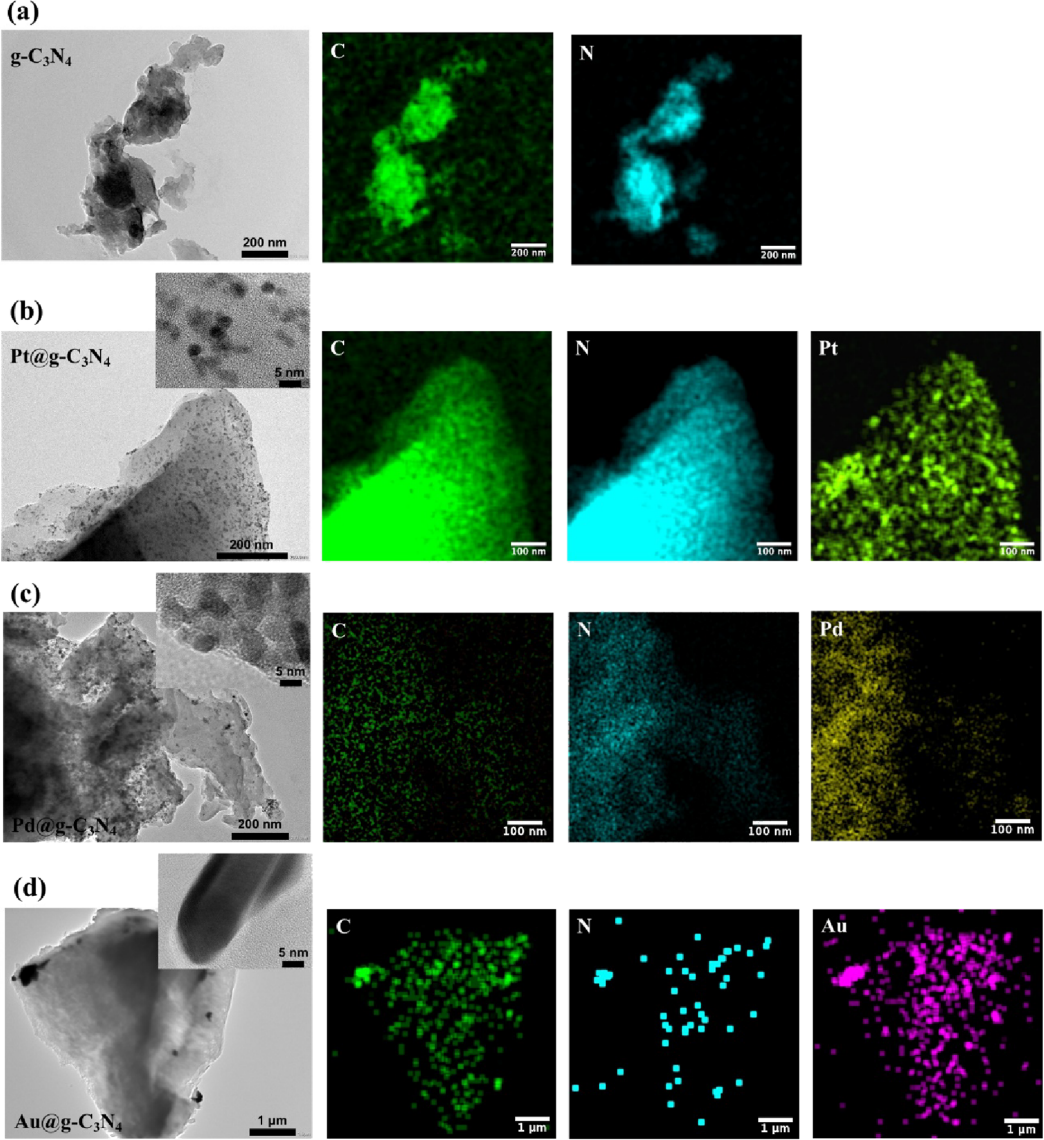
TEM images and EDX elemental mapping analysis of (a) g-C_3_N_4_, (b) Pt@g-C_3_N_4_, (c) Pd@g-C_3_N_4_, and (d) Au@g-C_3_N_4_.

To improve/modify the photocatalytic reactivity, metal co-catalysts can be employed. In particular, for the photoelectrochemical production of hydrogen, the presence of Pt nanoparticles was shown to be important and attributed to the noble metal-capturing photoexcited electrons during charge separation.^47^ Here, the effects of photogenerated nano-Pt, nano-Pd, and nano-Au attached to the g-C_3_N_4_ particles are evaluated for the production of H_2_O_2_. Figure 5 shows TEM images of (a) bare g-C_3_N_4_ and (b) nano-Pt-, (c) nano-Pd-, (d) nano-Au-modified g-C_3_N_4_. The morphology of g-C_3_N_4_ before and after metal deposition remains the same, showing a typical layered structure. Clearly, dark spots can be observed in Figure 5b,c, which are identified as metal nanoparticles with diameters of around 2–3 nm for Pt@g-C_3_N_4_ and Pd@g-C_3_N_4_. Energy dispersive X-ray (EDX) mapping analysis further confirmed the successful photochemical metal deposition on the g-C_3_N_4_ sheets. For the gold-modified g-C_3_N_4_, only bigger particles typically of 100 nm diameter are observed localized in edge regions. EDX analysis confirms gold on the g-C_3_N_4_ surface. Gold may nucleate less readily on the g-C_3_N_4_ surface, and this may lead to the formation of bigger nanoparticles. Analysis by PXRD (see Figure S5 in the Supporting Information) confirms successful photochemical metal deposition for Pt, Pd, and Au.

Table 2 summarizes data for H_2_O_2_ production, employing suspensions of g-C_3_N_4_ and co-catalyst-modified materials Pt@g-C_3_N_4_, Pd@g-C_3_N_4_, and Au@g-C_3_N_4_. For Pt- and Pd-modified g-C_3_N_4_, a loss of reactivity relative to g-C_3_N_4_ is observed. The production of H_2_O_2_ has been suggested to rely on the rapid formation of the 1,4-endoperoxide species on g-C_3_N_4_, which results in selectivity for the two-electron reduction of oxygen.^18^ The loading with metal co-catalyst can increase the charge separation process by allowing the transfer of photoexcited electrons from the g-C_3_N_4_ conduction band to the metal particles. Although charge separation may be improved, the production of H_2_O_2_ may be less effective with metal loading as endo-peroxides have to form directly on the g-C_3_N_4_ surface and not on the metal. This conclusion agrees with previous studies. A decrease in photoactivated 1,4-endoperoxide species was inferred from the EPR measurement for Pt@g-C_3_N_4_.^22^ Only Au@g-C_3_N_4_ exhibits significant H_2_O_2_ production reactivity in the presence of 100 mM glucose. Gold is known to (electro)chemically produce H_2_O_2_ from O_2_ at intermediate/mild reduction potentials.^70,71^ In fact, the presence of gold seems to double the yield of H_2_O_2_. However, considering the more complex preparation of Au@g-C_3_N_4_, the focus in this report remains on photocatalysis with pure g-C_3_N_4_ and without a co-catalyst.

**Table 2:** Comparison of Metal-Deposited g-C_3_N_4_ Performance for the Photogeneration of Hydrogen Peroxide^a^

catalyst	amount	reaction method	quencher	reaction time	H_2_O_2_ concentration
g-C_3_N_4_	5 mg	suspension	0.1 M glucose	1 h	66 ± 13 μM
Pt@g-C_3_N_4_	5 mg	suspension	0.1 M glucose	1 h	none
Pd@g-C_3_N_4_	5 mg	suspension	0.1 M glucose	1 h	none
Au@g-C_3_N_4_	5 mg	suspension	0.1 M glucose	1 h	138 ± 30 μM

a In 20 mL solution, suspension, 1 h, λ = 385 nm LED light, 80 mW cm^-2^. Errors estimated at ±20%.

### 3.4 Photogeneration of Hydrogen Peroxide IV: Effect of Triton X-100 Quencher

Next, the importance of binding hole quencher systems was further investigated by selecting the amphiphilic surfactant Triton X-100. Low concentrations of surface-active quencher material could be sufficient to help produce hydrogen peroxide. To explore the effects of g-C_3_N_4_ and PIM-1 in this photocatalytic reaction, three types of materials are compared: (i) g-C_3_N_4_ suspension, (ii) g-C_3_N_4_@PIM-1 immobilized on filter paper, and (iii) g-C_3_N_4_@PIM-1 particles (see the Experimental Section).

**Figure 6:**
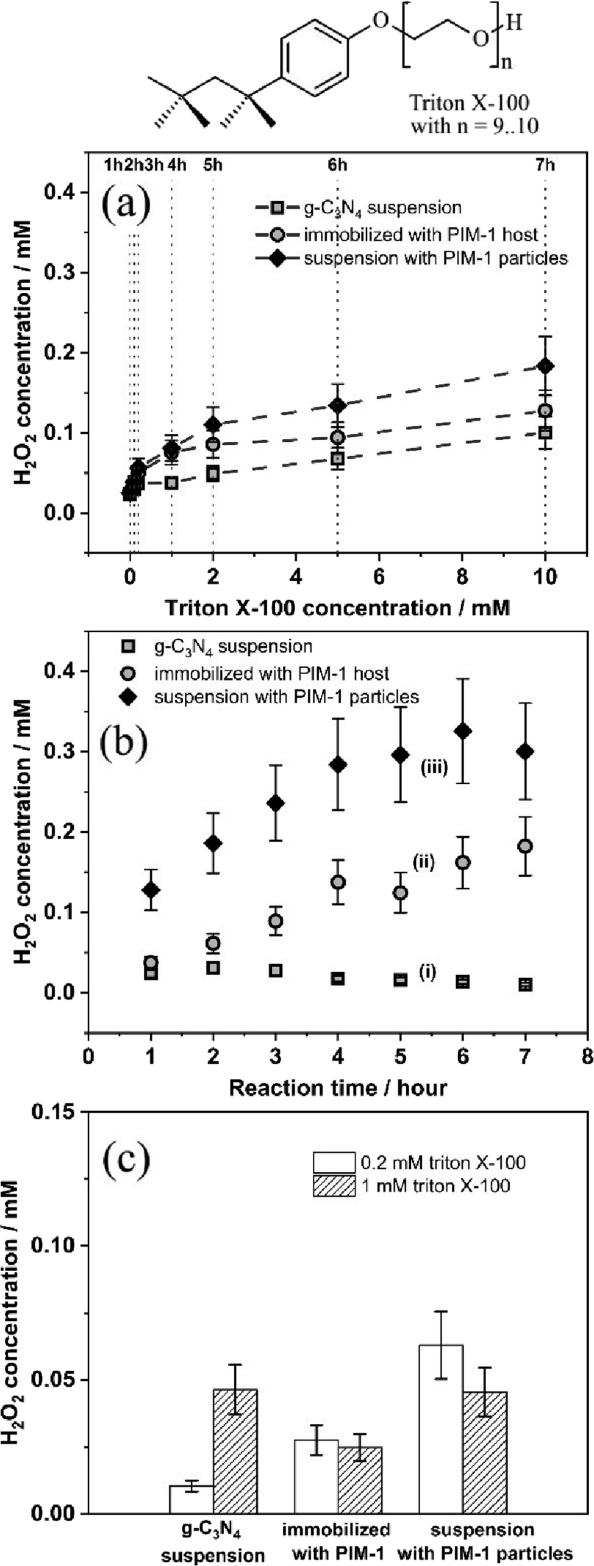
Molecular structure of Triton X-100 and (a) photogeneration of H_2_O_2_ with (i) 5 mg of g-C_3_N_4_ in suspension, (ii) 6 mg of g-C_3_N_4_@PIM-1 (containing 5 mg of g-C_3_N_4_) immobilized on 4 cm × 1 cm filter paper, and (iii) 6 mg of g-C_3_N_4_@PIM-1 particles (containing 5 mg of g-C_3_N_4_) in suspension (in 20 mL of solution; stepwise addition of Triton X-100; λ = 385 nm LED light). (b) Plot of H_2_O_2_ concentration versus reaction time for (i) 5 mg of g-C_3_N_4_ in suspension, (ii) 6 mg of g-C_3_N_4_@PIM-1 (containing 5 mg of g-C_3_N_4_) immobilized on 4 cm × 1 cm filter paper, and (iii) 6 mg of g-C_3_N_4_@PIM-1 particles (containing 5 of mg g-C_3_N_4_) in 1 mM Triton X-100. (c) Comparison of photocatalytic H_2_O_2_ production over (i) 5 mg of g-C_3_N_4_ in suspension, (ii) 6 mg of g-C_3_N_4_@PIM-1 (containing 5 mg of g-C_3_N_4_) immobilized on 4 cm × 1 cm filter paper, and (iii) 6 mg of g-C_3_N_4_@PIM-1 particles (containing 5 mg of g-C_3_N_4_) in suspension at two distinct Triton X-100 concentrations, below and above CMC concentration, after 1 h reaction time. Estimated error in all data points is ±20%.

Triton X-100 (see molecular structure in Figure 6) is a neutral polyethylene glycol-based surfactant with a CMC range of 0.22 to 0.24 mM.^72,73^ Data in Figure 6a suggest that at low concentrations of Triton X-100, H_2_O_2_ production occurs either with (i) g-C_3_N_4_ suspension, with (ii) immobilized g-C_3_N_4_ in a PIM-1 host, and with (iii) g-C_3_N_4_@PIM-1 particles. Figure 6b shows data for the H_2_O_2_ production as a function of time for 0.2 mM Triton X-100 in 20 mL of water. The presence of PIM-1 clearly improves the performance, and in particular, suspended g-C_3_N_4_@PIM-1 particles appear effective. Figure 2 shows SEM images for the g-C_3_N_4_@PIM-1 particles. The reactivity of the photocatalyst in the presence of PIM-1 is substantially higher. The g-C_3_N_4_@PIM-1 particles in suspension produce twice as much H_2_O_2_, and the onset of photochemical reactivity is low.

Data in Figure 6c displays the reactivity trends for two different concentrations of Triton X-100 after 1 h of illumination. Even with concentrations as low as 0.2 mM Triton X-100, the production of H_2_O_2_ is observed. With pure g-C_3_N_4_, only a Triton X-100 concentration higher than the CMC produces hydrogen peroxide possibly due to a lack of adsorption at lower concentrations. With g-C_3_N_4_@PIM-1 immobilized on filter paper and with g-C_3_N_4_@PIM-1 particles, an increase in the rate of H_2_O_2_ production is observed. For the 1 mM concentration of Triton X-100, the beneficial effects from PIM-1 are not obvious. Overall, the photocatalyst g-C_3_N_4_@PIM-1 in suspension appears to be the most effective system for both below- and above-CMC concentrations. This raises the question of whether the binding of Triton X-100 occurs directly to the g-C_3_N_4_ photocatalyst surface or alternatively into the PIM-1 as the microporous host.

### 3.5 Photogeneration of Hydrogen Peroxide V: Effect of Triton X-100 Adsorption to PIM-1

**Figure 7:**
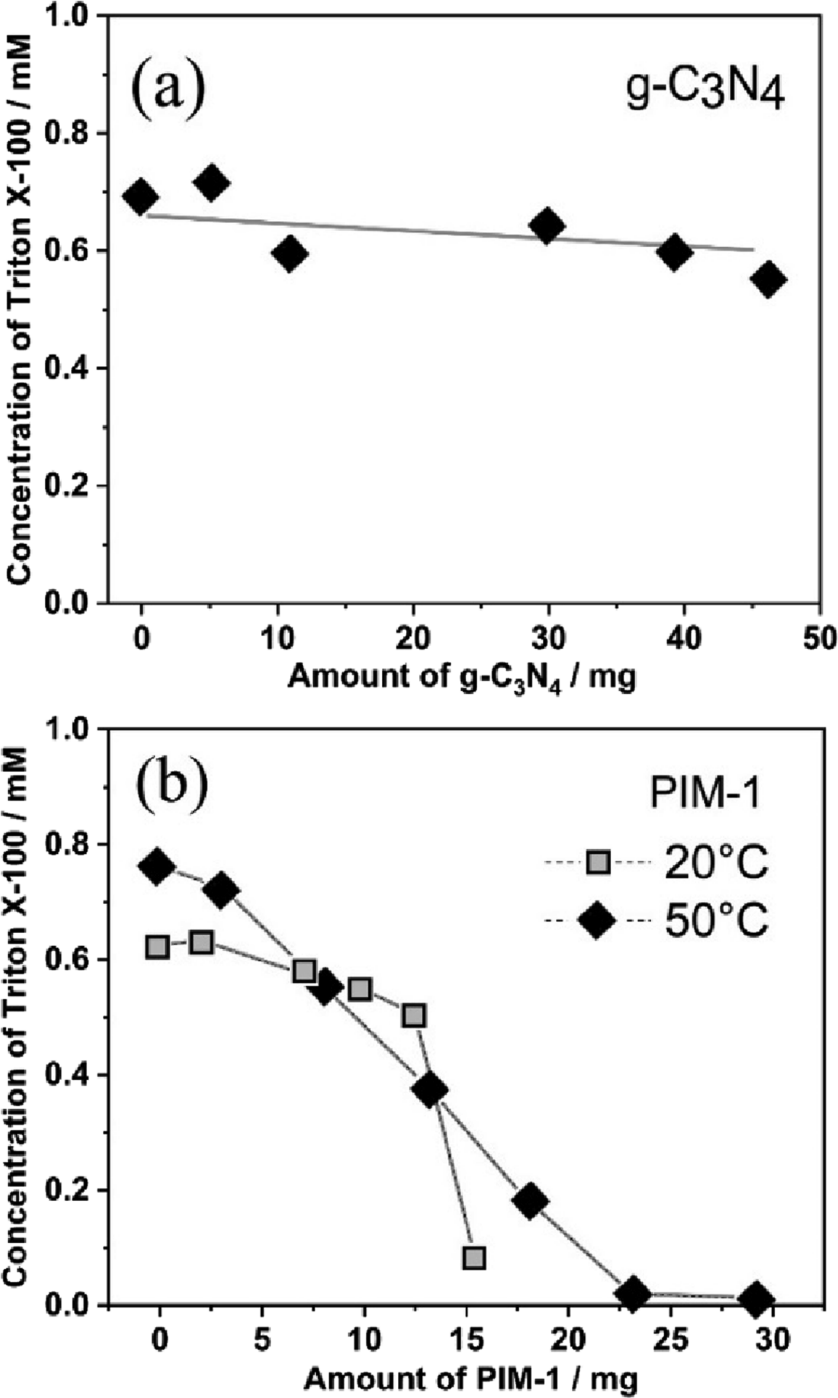
Binding experiment (monitored by ^1^H-NMR) for Triton X-100 in water solutions (initial volume, 15 mL; removal of 0.6 mL for each data point). (a) Plot of Triton X-100 concentration in solution versus g-C_3_N_4_ added. (b) Plot of Triton X-100 concentration in solution versus PIM-1 nanoparticle powder added (for both 20 and 50 °C). Trendlines added only as a guide to the eyes. Estimated error in all data points is ±20%.

To further study the ability of Triton X-100 to bind to g-C_3_N_4_ or to PIM-1, additional ^1^H-NMR experiments were performed. Figure 7 shows data based on monitoring the Triton X-100 concentrations with ^1^H-NMR when adding g-C_3_N_4_ (Figure 7a) and when adding PIM-1 particles (Figure 7b). When starting with a solution of 10.5 μmol in 15 mL (corresponding to a Triton X-100 concentration of approx. 0.7 mM), essentially no binding occurs with g-C_3_N_4_. Upon continued addition of g-C_3_N_4_, the solution concentration remains nearly constant. This could be linked to the insufficiently strong binding of Triton X-100 to the g-C_3_N_4_ surface. In contrast, data in Figure 7b suggest substantial interaction between the Triton X-100 and PIM-1 particles. The initial amount of 9.5 μmol in 15 mL of solution (corresponding to a Triton X-100 concentration of 0.6 mM) decreases essentially linearly with PIM-1 addition. The uptake is approx. one molecule of Triton X-100 for every four PIM-1 polymer repeat units (consistent with a water|PIM-1 partitioning process). Note that the partitioning process was slow at 20 °C but more clearly resolved at 50 °C. This is a substantial binding effect and a sign for effective quencher-filling of the microporous space (note that all data points are obtained in the range at or higher than the CMC,^68,69^ and therefore, a constant concentration-independent uptake of Triton X-100 seems likely). The hydrophobic nature of PIM-1 may be in part responsible for this accumulation of Triton X-100 into the microporous structure. PIM-1 is therefore able to bind Triton X-100 effectively, and this can lead to enhanced photochemical reactivity of g-C_3_N_4_ embedded into PIM-1.

## 4 Conclusions

It has been shown that adsorption (for both (i) onto the photocatalyst or (ii) into the microporous host) is an important step in the photocatalytic H_2_O_2_ production with g-C_3_N_4_. For glucose, adsorption of α-glucose (K_α-glucose_ = 200 ± 50 mol^–1^ dm^3^) is observed in ^1^H-NMR experiments with α-glucose binding being significantly stronger compared to β-glucose. In contrast, adsorption of glucose into PIM-1 was shown to be insignificant. Data for glucose-driven hydrogen peroxide production are therefore consistent with the binding of α-glucose to the photocatalyst before hole quenching processes are possible. In contrast, for Triton X-100, adsorption onto g-C_3_N_4_ was shown to be insignificant although Triton X-100 binding into PIM-1 is significant (with partitioning of one Triton X-100 molecule for every four PIM-1 monomeric repeat units). Production of hydrogen peroxide in the presence of Triton X-100 is enhanced in g-C_3_N_4_@PIM-1 (either immobilized in a film on filter paper or suspended as composite particles) when compared to bare g-C_3_N_4_. These are two distinct mechanistic cases with (i) adsorption directly onto the photocatalyst and (ii) adsorption indirectly into a host material with embedded photocatalysts.

The production of H_2_O_2_ is possible with suspended catalyst particles, but just as effective is the use of PIM-embedded photocatalyst immobilized, for example, on filter paper as substrate. The immobilized photocatalyst is easily fabricated and recoverable. The fact that PIM materials are molecularly rigid prevents them from directly interacting with the photocatalyst, although some photodegradation of PIM-1 and the resulting formation of H_2_O_2_ have been observed. In these preliminary experimental results, PIM-EA-TB represents a more photostable microporous polymer host. More experiments with PIM-EA-TB (and other types of PIMs) have to be performed in the future to provide a detailed comparison of adsorption effects and effects on photochemical reaction kinetics. Embedded into PIMs, photocatalyst surfaces are not obstructed and therefore able to interact with molecular quencher systems permeating/accumulating from solution into the microporous host. This study is exploratory in nature and may provide a starting point for the further development of photocatalysts in microporous PIM environments. The molecular structure of the PIM host will provide an opportunity to modify or enhance/tune photocatalytic activity.

## Supporting Information Available

Quantitative analysis of hydrogen peroxide concentration; binding assays with ^1^H-NMR; nitrogen-binding (BET) surface analysis; X-ray diffraction analysis; TEM and electron diffraction; Raman spectroscopy analysis; diffuse-reflectance UV/Vis data; XPS data (PDF)

## References

[ref1] Shu L.; Shi Y. Asymmetric Epoxidation using Hydrogen Peroxide (H_2_O_2_) as Primary Oxidant. Tetrahedron Lett. 1999, 40, 8721–8724. 10.1016/S0040-4039(99)01814-6.

[ref2] Zaharia C.; Suteu D.; Muresan A.; Muresan R.; Popescu A. Textile Wastewater Treatment by Homogenous Oxidation with Hydrogen Peroxide. Environ. Eng. Manage. J. 2009, 8, 1359–1369. 10.30638/eemj.2009.199.

[ref3] Boyce J. M. Modern Technologies for Improving Cleaning and Disinfection of Environmental Surfaces in Hospitals. Antimicrob. Resistance Infect. Control 2016, 5, 1–10. 10.1186/s13756-016-0111-x. PMC4827199 27069623

[ref4] Liu Y.; Zeng X.; Hu X.; Xia Y.; Zhang X. Solar-driven Photocatalytic Disinfection over 2D Semiconductors: The Generation and Effects of Reactive Oxygen Species. Solar RRL 2021, 5, 2000594 10.1002/solr.202000594.

[ref5] Wuorimaa A.; Jokela R.; Aksela R. Recent Developments in the Stabilization of Hydrogen Peroxide Bleaching of Pulps: An Overview. Nord. Pulp Pap. Res. J. 2006, 21, 435–443. 10.3183/npprj-2006-21-04-p435-443.

[ref6] Rai S.; Gupta T. P.; Shaki O.; Kale A. Hydrogen Peroxide: Its Use in an Extensive Acute Wound to Promote Wound Granulation and Infection Control–Is it better than normal Saline?. Int. J. Lower Extremity Wounds 2021, 15347346211032555 10.1177/15347346211032555. 34338578

[ref7] Ahmed A.; Hayat A.; John P.; Nawaz M. H.; Nasir M. Coral-shaped Tin Oxide Incorporated Graphitic Carbon Nitride Nanosheets as Peroxidase Mimic for Sensitive Colorimetric and Fluorescence Quenching Based Detection of Hydrogen Peroxide. J. Nanostruct. Chem. 2021, 675–691. 10.1007/s40097-021-00392-y.

[ref8] Martínez-Navarro F. J.; Martínez-Morcillo F. J.; de Oliveira S.; Candel S.; Cabas I.; García-Ayala A.; Martínez-Menchón T.; Corbalán-Vélez R.; Mesa-del-Castillo P.; Cayuela M. L.; Pérez-Oliva A. B.; García-Moreno D.; Mulero V. Hydrogen Peroxide in Neutrophil Inflammation: Lesson from the Zebrafish. Dev. Comp. Immunol. 2020, 105, 103583 10.1016/j.dci.2019.103583. 31862296

[ref9] Nitta Y.; Muraoka-Hirayama S.; Sakurai K. Catalase is Required for Peroxisome Maintenance during Adipogenesis. Biochim. Biophys. Acta, Mol. Cell Biol. Lipids 2020, 1865, 158726 10.1016/j.bbalip.2020.158726. 32335291

[ref10] Campos-Martin J. M.; Blanco-Brieva G.; Fierro J. L. G. Hydrogen Peroxide Synthesis: An Outlook Beyond the Anthraquinone Process. Angew. Chem., Int. Ed. 2006, 45, 6962–6984. 10.1002/anie.200503779. 17039551

[ref11] Ranganathan S.; Sieber V. Recent Advances in the Direct Synthesis of Hydrogen Peroxide using Chemical Catalysis - A review. Catalysts 2018, 8, 379 10.3390/catal8090379.

[ref12] Wang N.; Ma S.; Zuo P.; Duan J.; Hou B. Recent Progress of Electrochemical Production of Hydrogen Peroxide by Two-electron Oxygen Reduction Reaction. Adv. Sci. 2021, 8, 2100076 10.1002/advs.202100076. PMC8336511 34047062

[ref13] Ledendecker M.; Pizzutilo E.; Malta G.; Fortunato G. V.; Mayrhofer K. J. J.; Hutchings G. J.; Freakley S. J. Isolated Pd Sites as Selective Catalysts for Electrochemical and Direct Hydrogen Peroxide Synthesis. ACS Catal. 2020, 10, 5928–5938. 10.1021/acscatal.0c01305.

[ref14] Kumar A.; Raizada P.; Hosseini-Bandegharaei A.; Thakur V. K.; Nguyen V.-H.; Singh P. C-, N-Vacancy Defect Engineered Polymeric Carbon Nitride Towards Photocatalysis: Viewpoints and Challenges. J. Mater. Chem. A 2021, 9, 111–153. 10.1039/D0TA08384D.

[ref15] Dunand C.; Crèvecoeur M.; Penel C. Distribution of Superoxide and Hydrogen Peroxide in Arabidopsis Root and their Influence on Root Development: Possible Interaction with Peroxidases. New Phytol. 2007, 174, 332–341. 10.1111/j.1469-8137.2007.01995.x. 17388896

[ref16] Wang H.; Wan K.; Shi X. Recent Advances in Nanozyme Research. Adv. Mater. 2019, 31, 1805368 10.1002/adma.201805368. 30589120

[ref17] Shiraishi Y.; Kanazawa S.; Kofuji Y.; Sakamoto H.; Ichikawa S.; Tanaka S.; Hirai T. Sunlight-driven Hydrogen Peroxide Production from Water and Molecular Oxygen by Metal-free Photocatalysts. Angew. Chem., Int. Ed. 2014, 53, 13454–13459. 10.1002/anie.201407938. 25293501

[ref18] Boelrijk A. E. M.; Dismukes G. C. Mechanism of Hydrogen Peroxide Dismutation by a Dimanganese Catalase Mimic: Dominant Role of an Intramolecular Base on Substrate Binding Affinity and Rate Acceleration. Inorg. Chem. 2000, 39, 3020–3028. 10.1021/ic9911771. 11196896

[ref19] Hou H.; Zeng X.; Zhang X. Production of Hydrogen Peroxide by Photocatalytic Processes. Angew. Chem., Int. Ed. 2020, 59, 17356–17376. 10.1002/anie.201911609. 31571331

[ref20] Song H.; Wei L.; Chen L.; Zhang H.; Su J. Photocatalytic Production of Hydrogen Peroxide over Modified Semiconductor Materials: a Minireview. Top. Catal. 2020, 63, 895–912. 10.1007/s11244-020-01317-9.

[ref21] Shiraishi Y.; Kanazawa S.; Sugano Y.; Tsukamoto D.; Sakamoto H.; Ichikawa S.; Hirai T. Highly Selective Production of Hydrogen Peroxide on Graphitic Carbon Nitride (g-C_3_N_4_) Photocatalyst Activated by Visible Light. ACS Catal. 2014, 4, 774–780. 10.1021/cs401208c.

[ref22] Yamada Y.; Nomura A.; Miyahigashi T.; Ohkubo K.; Fukuzumi S. Acetate Induced Enhancement of Photocatalytic Hydrogen Peroxide Production from Oxalic Acid and Dioxygen. J. Phys. Chem. A 2013, 117, 3751–3760. 10.1021/jp312795f. 23631436

[ref23] Kormann C.; Bahnemann D. W.; Hoffmann M. R. Photocatalytic Production of H_2_O_2_ and Organic Peroxides in Aqueous Suspensions of TiO_2_, ZnO, and Desert Sand. Environ. Sci. Technol. 1988, 22, 798–806. 10.1021/es00172a009. 22195664

[ref24] aDong G.; Zhang Y.; Pan Q.; Qiu J. A Fantastic Graphitic Carbon Nitride (g-C_3_N_4_) Material: Electronic Structure, Photocatalytic and Photoelectronic Properties. J. Photochem. Photobiol., C 2014, 20, 33–50. 10.1016/j.jphotochemrev.2014.04.002.

[ref25] Wang Y.; Wang X.; Antonietti M. Polymeric Graphitic Carbon Nitride as a Heterogeneous Organocatalyst: From Photochemistry to Multipurpose Catalysis to Sustainable Chemistry. Angew. Chem., Int. Ed. 2012, 51, 68–89. 10.1002/anie.201101182. 22109976

[ref26] Goettmann F.; Fischer A.; Antonietti M.; Thomas A. Chemical Synthesis of Mesoporous Carbon Nitrides using Hard Templates and their Use as a Metal-free Catalyst for Friedel–Crafts Reaction of Benzene. Angew. Chem., Int. Ed. 2006, 45, 4467–4471. 10.1002/anie.200600412. 16770823

[ref27] Goettmann F.; Fischer A.; Antonietti M.; Thomas A. Metal-free Catalysis of Sustainable Friedel–Crafts Reactions: Direct Activation of Benzene by Carbon Nitrides to Avoid the Use of Metal Chlorides and Halogenated Compounds. Chem. Commun. 2006, 4530–4532. 10.1039/B608532F. 17283808

[ref28] Wang X.; Maeda K.; Thomas A.; Takanabe K.; Xin G.; Carlsson J. M.; Domen K.; Antonietti M. A Metal-free Polymeric Photocatalyst for Hydrogen Production from Water under Visible Light. Nat. Mater. 2009, 8, 76–80. 10.1038/nmat2317. 18997776

[ref29] Truong H. B.; Bae S.; Cho J.; Hur J. Advances in Application of g–C_3_N_4_–based Materials for Treatment of Polluted Water and Wastewater via Activation of Oxidants and Photoelectrocatalysis: A Comprehensive Review. Chemosphere 2022, 131737 10.1016/j.chemosphere.2021.131737. 34352551

[ref30] Li X.; Huang G.; Chen X.; Huang J.; Li M.; Yin J.; Liang Y.; Yao Y.; Li Y. A Review on Graphitic Carbon Nitride (g-C_3_N_4_) Based Hybrid Membranes for Water and Wastewater Treatment. Sci. Total Environ. 2021, 148462 10.1016/j.scitotenv.2021.148462. 34465053

[ref31] Zhu Q.; Xu Z.; Qiu B.; Xing M.; Zhang J. Emerging Cocatalysts on g-C_3_N_4_ for Photocatalytic Hydrogen Evolution. Small 2021, 17, 2101070 10.1002/smll.202101070. 34318978

[ref32] Malik R.; Tomer V. K. State-of-the-art Review of Morphological Advancements in Graphitic Carbon Nitride (g-CN) for Sustainable Hydrogen Production. Renewable Sustainable Energy Rev. 2021, 135, 110235 10.1016/j.rser.2020.110235.

[ref33] Fronczak M.; Krajewska M.; Demby K.; Bystrzejewski M. Extraordinary Adsorption of Methyl Blue onto Sodium-doped Graphitic Carbon Nitride. J. Phys. Chem. C 2017, 121, 15756–15766. 10.1021/acs.jpcc.7b03674.

[ref34] Tan J.; Tian N.; Li Z.; Li J.; Yao X.; Vakili M.; Lu Y.; Zhang T. Intrinsic Defect Engineering in Graphitic Carbon Nitride for Photocatalytic Environmental Purification: A Review to Fill Existing Knowledge Gaps. Chem. Eng. J. 2021, 127729 10.1016/j.cej.2020.127729.

[ref35] Bai L.; Huang H.; Yu S.; Zhang D.; Huang H.; Zhang Y. Role of Transition Metal Oxides in g-C_3_N_4_-based Heterojunctions for Photocatalysis and Supercapacitors. J. Energy Chem. 2022, 64, 214–235. 10.1016/j.jechem.2021.04.057.

[ref36] Liao G.; Gong Y.; Zhang L.; Gao H.; Yang G.-J.; Fang B. Semiconductor Polymeric Graphitic Carbon Nitride Photocatalysts: the ″Holy Grail″ for the Photocatalytic Hydrogen Evolution Reaction under Visible Light. Energy Environ. Sci. 2019, 12, 2080–2147. 10.1039/C9EE00717B.

[ref37] Warshagha M. Z. A.; Muneer M. Synthesis of Ph-modified Graphitic Carbon Nitride for Degradation of Different Chromophoric Organic Pollutants in Aqueous Suspension under Visible Light. Langmuir 2020, 36, 9719–9727. 10.1021/acs.langmuir.0c01055. 32787064

[ref38] Wang Y.; Liu L.; Ma T.; Zhang Y.; Huang H. 2D Graphitic Carbon Nitride for Energy Conversion and Storage. Adv. Funct. Mater. 2021, 31, 2102540 10.1002/adfm.202102540.

[ref39] Zhao Y.; Al Abass N. A.; Malpass-Evans R.; Carta M.; McKeown N. B.; Madrid E.; Fletcher P. J.; Marken F. Photoelectrochemistry of Immobilised Pt@g-C_3_N_4_ Mediated by Hydrogen and Enhanced by a Polymer of Intrinsic Microporosity PIM-1. Electrochem. Commun. 2019, 103, 1–6. 10.1016/j.elecom.2019.04.006.

[ref40] Fu J.; Yu J.; Jiang C.; Cheng B. g-C_3_N_4_-based Heterostructured Photocatalysts. Adv. Energy Mater. 2018, 8, 1701503 10.1002/aenm.201701503.

[ref41] Cao Q.; Kumru B.; Antonietti M.; Schmidt B. V. K. J. Graphitic Carbon Nitride and Polymers: a Mutual Combination for Advanced Properties. Mater. Horiz. 2020, 7, 762–786. 10.1039/C9MH01497G.

[ref42] Zhao Y.; Dobson J.; Harabajiu C.; Madrid E.; Kanyanee T.; Lyall C.; Reeksting S.; Carta M.; McKeown N. B.; Torrente-Murciano L.; Black K.; Marken F. Indirect Photo-electrochemical Detection of Carbohydrates with Pt@g-C_3_N_4_ Immobilised into a Polymer of Intrinsic Microporosity (PIM-1) and Attached to a Palladium Hydrogen Capture Membrane. Bioelectrochemistry 2020, 134, 107499 10.1016/j.bioelechem.2020.107499. 32179453

[ref43] Su K.; Deng S.; Li L.; Qin Q.; Yang J.; Chen Y.; Zhang S.; Chen J. g-C_3_N_4_ Derived Materials for Photocatalytic Hydrogen Production: a Mini Review on Design Strategies. J. Renewable Mater. 2022, 10, 653–663. 10.32604/jrm.2022.018556.

[ref44] Liu M.; Wen Y.; Lu L.; Chen Y.; Tian X.; Jin H.; Liu J.; Dai K. Nitrogen-doped Graphene/Graphitic Carbon Nitride with Enhanced Charge Separation and Two-electron-transferring Reaction Activity for Boosting Photocatalytic Hydrogen Peroxide Production. Sustainable Energy Fuels 2021, 5, 1511–1520. 10.1039/D0SE01828G.

[ref45] Wu S.; Yu H.; Chen S.; Quan X. Enhanced Photocatalytic H_2_O_2_ Production over Carbon Nitride by Doping and Defect Engineering. ACS Catal. 2020, 10, 14380–14389. 10.1021/acscatal.0c03359.

[ref46] Wang Y.; Ghanem B. S.; Ali Z.; Hazazi K.; Han Y.; Pinnau I. Recent Progress on Polymers of Intrinsic Microporosity and Thermally Modified Analogue Materials for Membrane-based Fluid Separations. Small Struct. 2021, 2, 2100049 10.1002/sstr.202100049.

[ref47] Budd P. M.; Ghanem B. S.; Makhseed S.; McKeown N. B.; Msayib K. J.; Tattershall C. E. Polymers of Intrinsic Microporosity (PIMs): Robust, Solution-processable, Organic Nanoporous Materials. Chem. Commun. 2004, 2, 230–231. 10.1039/B311764B. 14737563

[ref48] McKeown N. B.; Budd P. M. Polymers of Intrinsic Microporosity (PIMs): Organic Materials for Membrane Separations, Heterogeneous Catalysis and Hydrogen Storage. Chem. Soc. Rev. 2006, 35, 675–683. 10.1039/b600349d. 16862268

[ref49] Budd P. M.; McKeown N. B.; Ghanem B. S.; Msayib K. J.; Fritsch D.; Starannikova L.; Belov N.; Sanfirova O.; Yampolskii Y.; Shantarovich V. Gas Permeation Parameters and other Physicochemical Properties of a Polymer of Intrinsic Microporosity: Polybenzodioxane PIM-1. J. Membr. Sci. 2008, 325, 851–860. 10.1016/j.memsci.2008.09.010.

[ref50] Marken F.; Wang L.; Zhao Y.; Li Z.; Amiri M.; Imanzadeh H. Polymers of Intrinsic Microporosity (PIMs) in Sensing and in Electroanalysis. Curr. Opin. Chem. Eng. 2022, 35, 100765 10.1016/j.coche.2021.100765.

[ref51] Li Z.; Malpass-Evans R.; McKeown N. B.; Carta M.; Mathwig K.; Lowe J. P.; Marken F. Effective Electroosmotic Transport of Water in an Intrinsically Microporous Polyamine (PIM-EA-TB). Electrochem. Commun. 2021, 130, 107110 10.1016/j.elecom.2021.107110.

[ref52] Wang L.; Zhao Y.; Fan B.; Carta M.; Malpass-Evans R.; McKeown N. B.; Marken F. Polymer of Intrinsic Microporosity (PIM) Films and Membranes in Electrochemical Energy Storage and Conversion: a Mini-review. Electrochem. Commun. 2020, 118, 106798 10.1016/j.elecom.2020.106798.

[ref53] Marken F.; Carta M.; McKeown N. B. Polymers of Intrinsic Microporosity in the Design of Electrochemical Multicomponent and Multiphase Interfaces. Anal. Chem. 2021, 93, 1213–1220. 10.1021/acs.analchem.0c04554. 33369401

[ref54] He D.; He D. S.; Yang J.; Low Z.-X.; Malpass-Evans R.; Carta M.; McKeown N. B.; Marken F. Molecularly Rigid Microporous Polyamine Captures and Stabilizes Conducting Platinum Nanoparticle Networks. ACS Appl. Mater. Interfaces 2016, 8, 22425–22430. 10.1021/acsami.6b04144. 27509837

[ref55] Zhao Y.; Malpass-Evans R.; Carta M.; McKeown N. B.; Fletcher P. J.; Kociok-Köhn G.; Lednitzky D.; Marken F. Size-selective Photoelectrochemical Reactions in Microporous Environments: Clark Probe Investigation of Pt@g-C_3_N_4_ Embedded into Intrinsically Microporous Polymer (PIM-1). ChemElectroChem 2021, 8, 3499–3505. 10.1002/celc.202100732.

[ref56] Budd P. M.; Elabas E. S.; Ghanem B. S.; Makhseed S.; McKeown N. B.; Msayib K. J.; Tattershall C. E.; Wang D. Solution-processed, Organophilic Membrane Derived from a Polymer of Intrinsic Microporosity. Adv. Mater. 2004, 16, 456–459. 10.1002/adma.200306053.

[ref57] Carta M.; Malpass-Evans R.; Croad M.; Rogan Y.; Jansen J. C.; Bernardo P.; Bazzarelli F.; McKeown N. B. An Efficient Polymer Molecular Sieve for Membrane Gas Separations. Science 2013, 339, 303–307. 10.1126/science.1228032. 23329042

[ref58] Yan S. C.; Li Z. S.; Zou Z. G. Photodegradation Performance of g-C_3_N_4_ Fabricated by Directly Heating Melamine. Langmuir 2009, 25, 10397–10401. 10.1021/la900923z. 19705905

[ref59] Liu M.; Xia P.; Zhang L.; Cheng B.; Yu J. Enhanced Photocatalytic H_2_-Production Activity of g-C_3_N_4_ Nanosheets via Optimal Photodeposition of Pt as Cocatalyst. ACS Sustainable Chem. Eng. 2018, 6, 10472–10480. 10.1021/acssuschemeng.8b01835.

[ref60] Madrid E.; Lowe J. P.; Msayib K. J.; McKeown N. B.; Song Q.; Attard G. A.; Duren T.; Marken F. Triphasic Nature of Polymers of Intrinsic Microporosity Induces Storage and Catalysis Effects in Hydrogen and Oxygen Reactivity at Electrode Surfaces. ChemElectroChem 2019, 6, 252–259. 10.1002/celc.201800177.

[ref61] Wang L.; Carta M.; Malpass-Evans R.; McKeown N. B.; Fletcher P. J.; Lednitzky D.; Marken F. Hydrogen Peroxide versus Hydrogen Generation at Bipolar Pd/Au Nano-catalysts Grown into an Intrinsically Microporous Polyamine (PIM-EA-TB). Electrocatalysis 2021, 12, 771–784. 10.1007/s12678-021-00692-5.

[ref62] Köwitsch I.; Mehring M. Carbon Nitride Materials: Impact of Synthetic Method on Photocatalysis and Immobilization for Photocatalytic Pollutant Degradation. J. Mater. Sci. 2021, 56, 18608–18624. 10.1007/s10853-021-06405-z.

[ref63] Jürgens B.; Irran E.; Senker J.; Kroll P.; Müller H.; Schnick W. Melem (2,5,8-triamino-tri-s-triazine), an Important Intermediate During Condensation of Melamine Rings to Graphitic Carbon Nitride: Synthesis, Structure Determination by X-ray Powder Diffractometry, Solid-state NMR, and Theoretical Studies. J. Am. Chem. Soc. 2003, 125, 10288–10300. 10.1021/ja0357689. 12926953

[ref64] Sun J. X.; Yuan Y. P.; Qiu L. G.; Jiang X.; Xie A. J.; Shen Y. H.; Zhu J. F. Fabrication of Composite Photocatalyst g-C_3_N_4_-ZnO and Enhancement of Photocatalytic Activity under Visible Light. Dalton Trans. 2012, 41, 6756–6763. 10.1039/c2dt12474b. 22532247

[ref65] Zinin P. V.; Ming L.-C.; Sharma S. K.; Khabashesku V. N.; Liu X.; Hong S.; Endo S.; Acosta T. Ultraviolet and Near-infrared Raman Spectroscopy of Graphitic C_3_N_4_ Phase. Chem. Phys. Lett. 2009, 472, 69–73. 10.1016/j.cplett.2009.02.068.

[ref66] Song Q.; Cao S.; Zavala-Rivera P.; Lu L. P.; Li W.; Ji Y.; Al-Muhtaseb S. A.; Cheetham A. K.; Sivaniah E. Photo-oxidative Enhancement of Polymeric Molecular Sieve Membranes. Nat. Commun. 2013, 4, 1–9. 10.1038/ncomms2942. 23715277

[ref67] Ray P.; Gidley D.; Badding J. V.; Lueking A. D. UV and Chemical Modifications of Polymer of Intrinsic Microporosity 1 to Develop Vibrational Spectroscopic Probes of Surface Chemistry and Porosity. Microporous Mesoporous Mater. 2019, 277, 29–35. 10.1016/j.micromeso.2018.09.013.

[ref68] Liang J.; Deng J. A Chiral Interpenetrating Polymer Network Constructed by Helical Substituted Polyacetylenes and Used for Glucose Adsorption. Polym. Chem. 2017, 8, 1426–1434. 10.1039/C7PY00025A.

[ref69] Olsson R.; Giesler R.; Persson P. Adsorption Mechanisms of Glucose in Aqueous Goethite Suspensions. J. Colloid Interface Sci. 2011, 353, 263–268. 10.1016/j.jcis.2010.09.023. 20933242

[ref70] Mitsutaka O.; Yasutaka K.; Kizashi Y.; Tomoki A.; Susumu T.; Masatake H. Direct Production of Hydrogen Peroxide from H_2_ and O_2_ over Highly Dispersed Au Catalysts. Chem. Lett. 2003, 32, 822–823. 10.1246/cl.2003.822.

[ref71] Jirkovský J. S.; Halasa M.; Schiffrin D. J. Kinetics of Electrocatalytic Reduction of Oxygen and Hydrogen Peroxide on Dispersed Gold Nanoparticles. Phys. Chem. Chem. Phys. 2010, 12, 8042–8052. 10.1039/c002416c. 20505889

[ref72] Morandat S.; El Kirat K. Membrane Resistance to Triton X-100 Explored by Real-time Atomic Force Microscopy. Langmuir 2006, 22, 5786–5791. 10.1021/la0604228. 16768509

[ref73] Koley D.; Bard A. J. Triton X-100 Concentration Effects on Membrane Permeability of a Single HeLa Cell by Scanning Electrochemical Microscopy (SECM). Proc. Natl. Acad. Sci. 2010, 107, 16783–16787. 10.1073/pnas.1011614107. 20837548 PMC2947864

