# Supplementary material for: Effects of g-C3N4 Heterogenization into Intrinsically Microporous Polymers on the Photocatalytic Generation of Hydrogen Peroxide
Source: ACS Appl Mater Interfaces. 2022 Apr 24;14(17):19938–48. doi: 10.1021/acsami.1c23960 (PMC9073839; doi:10.1021/acsami.1c23960)
Supplement: Supplementary file 1 — am1c23960_si_001.pdf [file am1c23960_si_001.pdf]

Supporting Information

Effects of g-C_3N_4 Heterogenization into Intrinsically Microporous Polymers on the Photocatalytic Generation of Hydrogen Peroxide

Yuanzhu Zhao [1], Lina Wang [1], Richard Malpass-Evans [2], Neil B. McKeown [2], Mariolino Carta [3], John P. Lowe [4], Catherine L. Lyall [4], Rémi Castaing [4], Philip J. Fletcher [4], Gabriele Kociok-Köhn [4], Jannis Wenk [5], Zhenyu Guo [6], and Frank Marken *[1]

[1] *Department of Chemistry, University of Bath, Claverton Down, Bath BA2 7AY, UK*
[2] *EaStCHEM School of Chemistry, University of Edinburgh, Joseph Black Building, David Brewster Road, Edinburgh, Scotland EH9 3JF, UK*
[3] *Department of Chemistry, Swansea University, College of Science, Grove Building, Singleton Park, Swansea SA2 8PP, UK*
[4] *University of Bath, Materials & Chemical Characterisation Facility, MC[2], Bath BA2 7AY, UK*
[5] *Department of Chemical Engineering and Water Innovation Research Centre, WIRC, University of Bath, Claverton Down, Bath BA2 7AY, UK*
[6] *Department of Chemical Engineering, Imperial College London, South Kensington Campus London, SW7 2AZ, UK*

Corresponding Author:

Email: f.marken@bath.ac.uk

Content

(I) Quantitative analysis of hydrogen peroxide concentration 3

Figure S1. Reaction of H_2O_2 with para-nitrophenyl boronic acid to give para-nitrophenol.

(II) Binding assays with ^{1}H-NMR 3

Figure S2. ^{1}H NMR spectrum for glucose in water solution with an internal standard molecule DMSO.
Figure S3. ^{1}H NMR spectrum for Triton X-100 in water solution with an internal standard molecule DMSO.
Table S1. ^{1}H-NMR chemical shift data for alpha glucose, beta glucose, and for Triton X-100 in H_2O solutions employed for concentration determination.

(III) Nitrogen binding (BET) surface analysis 7

Figure S4. N_2 adsorption isotherm and pore size distribution for (a) graphitic carbon nitride and (b) PIM-1 powders. For g-C_3N_4 BJH pore size calculation was used with cylindric pores and for PIM-1 a QSDFT pore size calculation with cylindric pores was employed.

(IV) X-ray diffraction analysis 8

Figure S5. Powder X-ray diffraction (PXRD) pattern of pure graphitic carbon nitride (black), graphitic carbon nitride with platinum deposition (red), palladium deposition (blue) and gold deposition (green).

(V) Transmission electron microscopy (TEM) analysis 9
Figure S6. Transmission electron microscopy (TEM) images (A,B) and electron diffraction (C) data for pure g-C_3N_4.

(VI) Raman spectroscopy analysis 10

Figure S7. Raman spectrum obtained at 325 nm excitation for g-C_3N_4. Background subtracted.

(VII) Diffuse-reflectance UV/Vis spectrum 11

Figure S8. Diffuse-reflectance spectrum for g-C_3N_4. (A) Background subtracted reflectance data. (B) Tauc plot based on Kubelka-Munk transform.

(VIII) X-ray photoelectron spectroscopy (XPS) analysis 11

Figure S9. (a) XPS survey spectra of g-C_3N_4. (b) Higher resolution data for C1s of g-C_3N_4. (c) Higher resolution data for N1s of g-C_3N_4.
Table S2. XPS data analysis.

(I) Quantitative analysis of hydrogen peroxide concentration. This methodology follows an indirect detection method from literature[1] based on the reaction of 4-nitrophenylboronic acid to 4-nitrophenol. Typically, 4-nitrophenylboronic acid (Sigma Aldrich) was dissolved in DMSO with a concentration of 10 mM. Then 100 μL prepared 4- nitrophenylboronic acid solution was added to a 10 mL mixing solution of 10% DMSO and 90% 10mM carbonate buffer (pH 9) and stocked as a detection reagent solution. After photochemical experiments, 100 μL sample solution was taken from the vial and mixed with 1mL detection reagent solution. After leaving in a totally dark environment for 1 hour to allow complete reaction, the reacted solution is diluted by 10 times with a mixing solution of 10% DMSO and 90% H_2O and went to the mass spectrometry measurement. By quantitively analysing the peaks of nitrophenol from mass spectrometry, the corresponding amount of H_2O_2 contributed to the reaction can be calculated.

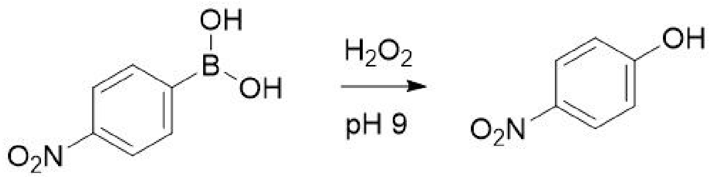

Figure S1. Reaction of H_2O_2 with *para*-nitrophenyl boronic acid to give *para*-nitrophenol.

(II) Binding assays with ^{1}H-NMR. Glucose and triton X-100 binding experiment. Initially, a water solution with approx. 1 mM glucose/ TritonX-10 and certain amount of DMSO as an internal standard molecule was prepared. A volume of 0.6 mL solution was extracted to an NMR tube for every measurement. Certain amount (typically 5 mg) g-C_3N_4/ PIM-1 powder was added into the solution and stirred at room temperature (or at elevated temperature) for 20 mins to allow sufficient adsorption of glucose/ Triton X-100 on the material (g-C_3N_4/ PIM-1 powder). Binding was followed by NMR experiments. Detailed calculations refer to the NMR spectra below and Table S1.

NMR spectra:

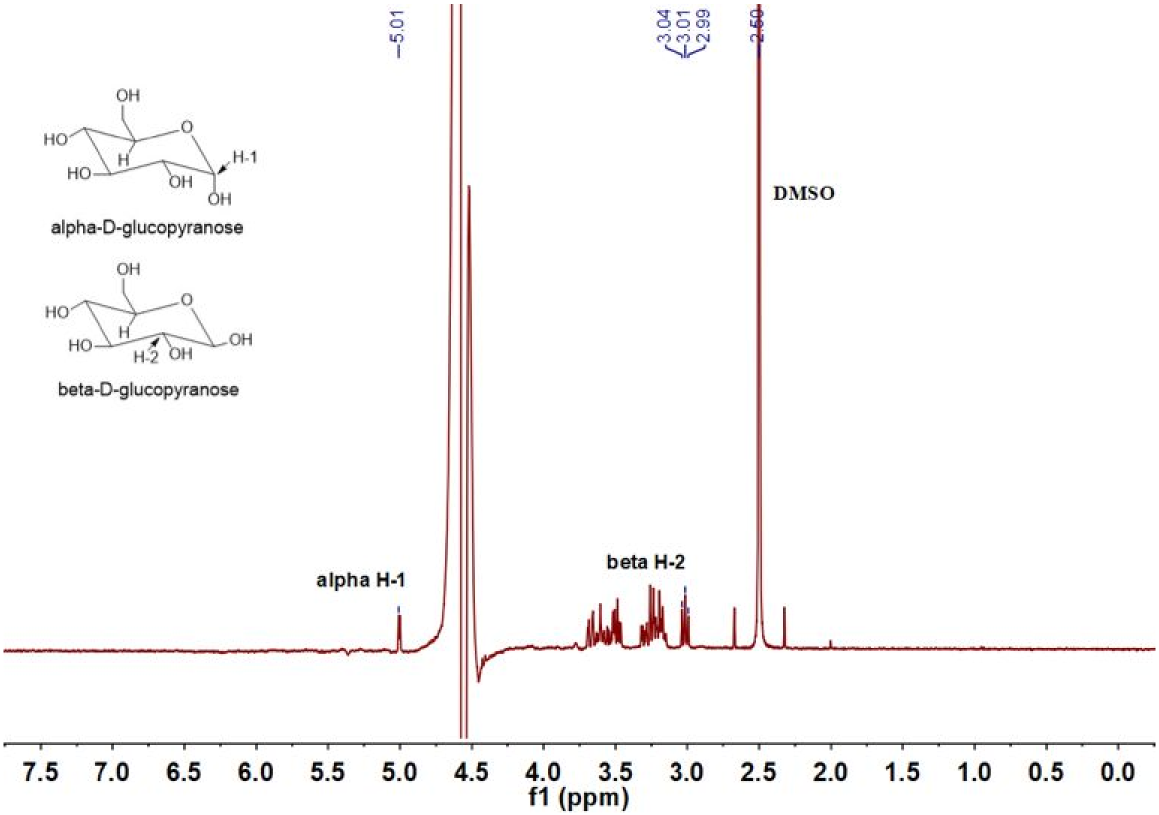

Figure S2. ^{1}H NMR spectrum for glucose in water solution with an internal standard molecule DMSO.

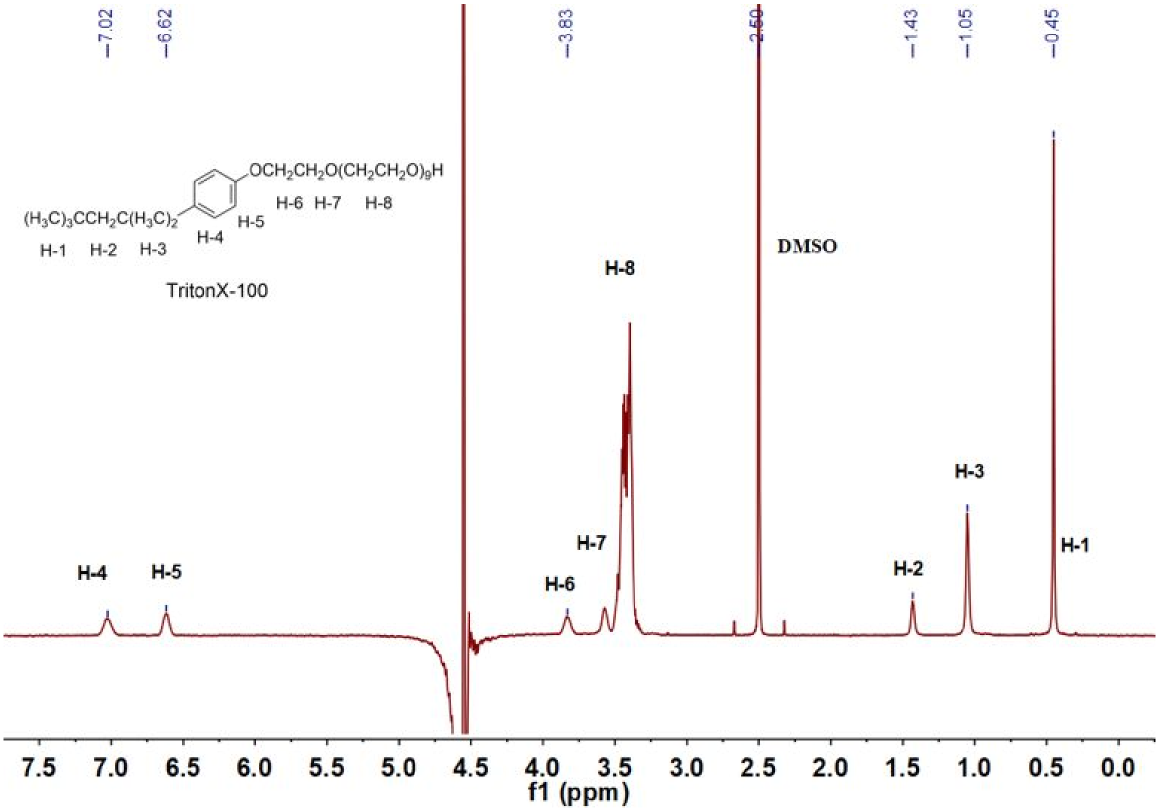

Figure S3. ^{1}H NMR spectrum for Triton X-100 in water solution with an internal standard molecule DMSO.

Table S1. ^{1}H-NMR chemical shift data for α-glucose, β-glucose, and for Triton X-100 in H_2O solutions employed for concentration determination.

	H atom	^{1}H-NMR (ppm)	number of H atoms
α-glucose	H-1	5.01	1
β-glucose	H-2	3.01	1
Triton X-100	H-4	7.02	2
DMSO		2.50	6

To fit the binding assay data with Langmurian adsorption theory, the concentration of both α-glucose and β-glucose for each sample can be modelled in an Excel spreadsheet. The BET surface area (Figure S4) and an assumed binding area of 12.7×10^{-20} m^2 were employed. The theory lines for either α-glucose and β-glucose in Figure 4 are modelled with surface coverage θ equations below.

$$\theta_\alpha = \frac{K_\alpha C_\alpha}{1 + K_\alpha C_\alpha + K_\beta C_\beta} \tag{1}$$

$$\theta_\beta = \frac{K_\beta C_\beta}{1 + K_\alpha C_\alpha + K_\beta C_\beta} \tag{2}$$

with

K_α the binding constant for α-glucose ($mol^{-1}dm^3$)

K_β the binding constant for β-glucose ($mol^{-1}dm^3$)

C_α the concentration of α-glucose in the solution ($mol\,dm^{-3}$)

C_β the concentration of β-glucose in the solution ($mol\,dm^{-3}$).

(III) Nitrogen adsorption isotherm (BET) surface analysis

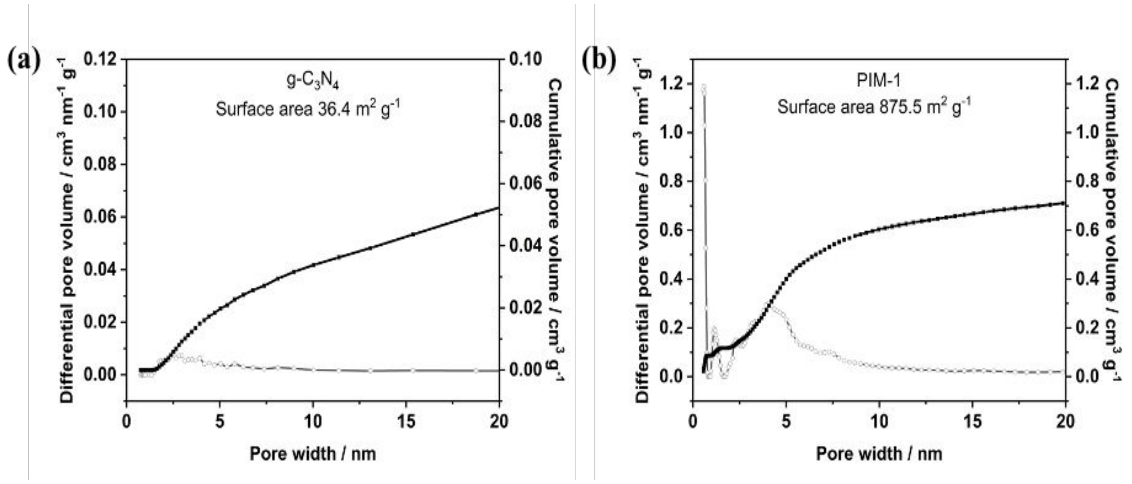

Figure S4. N_2 adsorption isotherm and pore size distribution for (a) graphitic carbon nitride and (b) PIM-1 powders. For g-C_3N_4 BJH pore size calculation was used with cylindric pores and for PIM-1 a QSDFT pore size calculation with cylindric pores was employed.

(IV) X-ray diffraction analysis

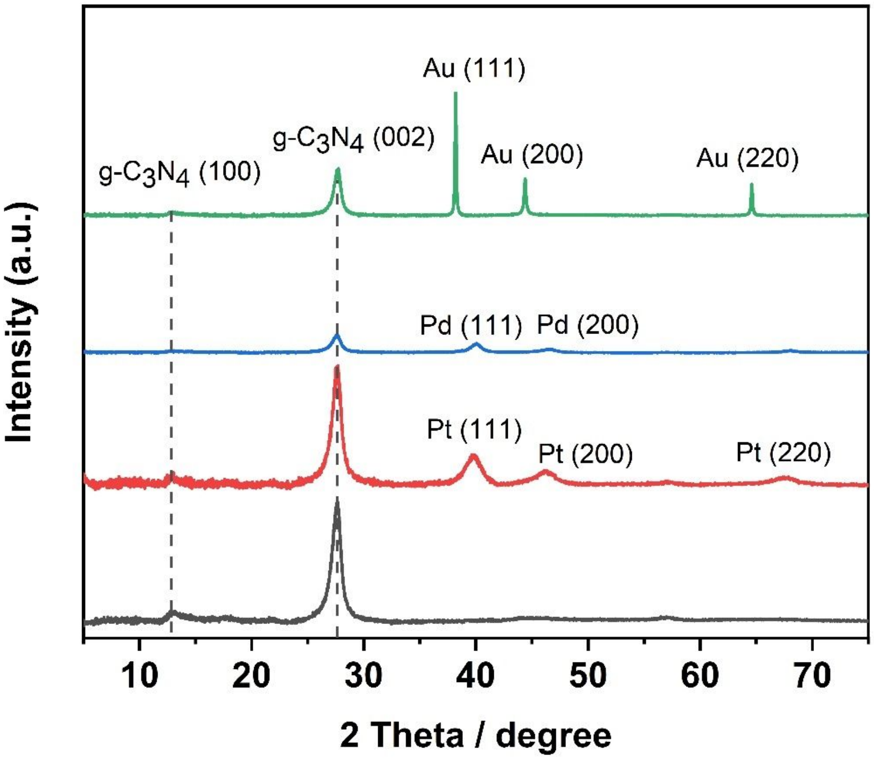

Figure S5. Powder X-ray diffraction (PXRD) pattern of pure graphitic carbon nitride (black), graphitic carbon nitride with platinum deposition (red), palladium deposition (blue) and gold deposition (green).

(V) Transmission electron microscopy (TEM) analysis

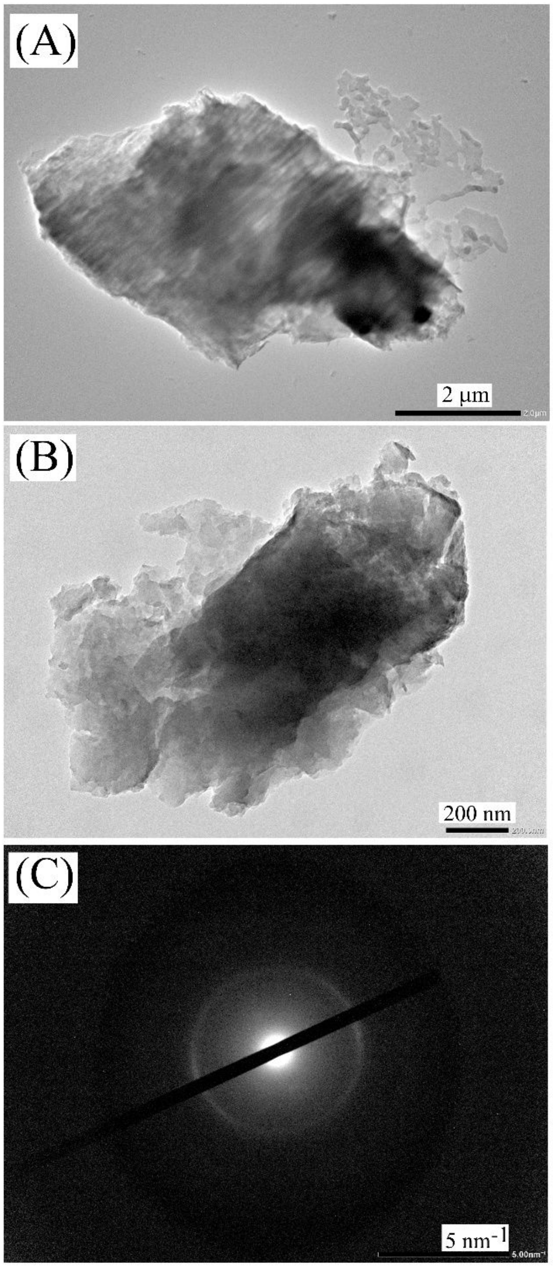

Figure S6. Transmission electron microscopy (TEM) images (A,B) and electron diffraction (C) data for pure g-C_3N_4.

(VI) Raman spectroscopy analysis

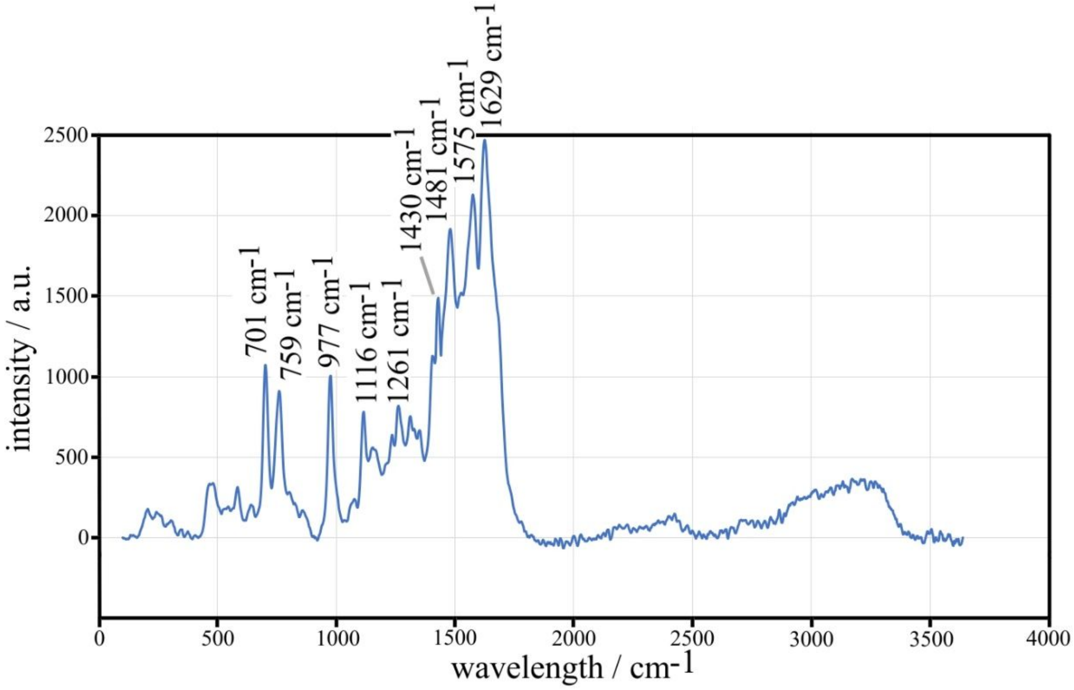

Figure S7. Raman spectrum obtained at 325 nm excitation for g-C_3N_4. Background subtracted.

(VII) Diffuse-reflectance UV/Vis spectrum

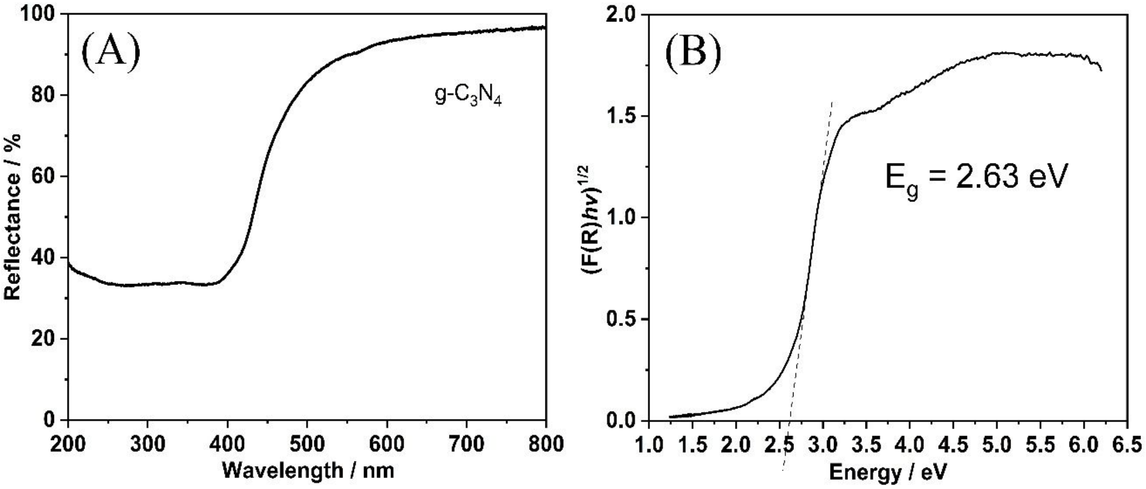

Figure S8. Diffuse-reflectance spectrum for g-C_3N_4. (A) Background subtracted reflectance data. (B) Tauc plot based on Kubelka-Munk transform [2].

(VIII) X-ray photoelectron spectroscopy (XPS) analysis

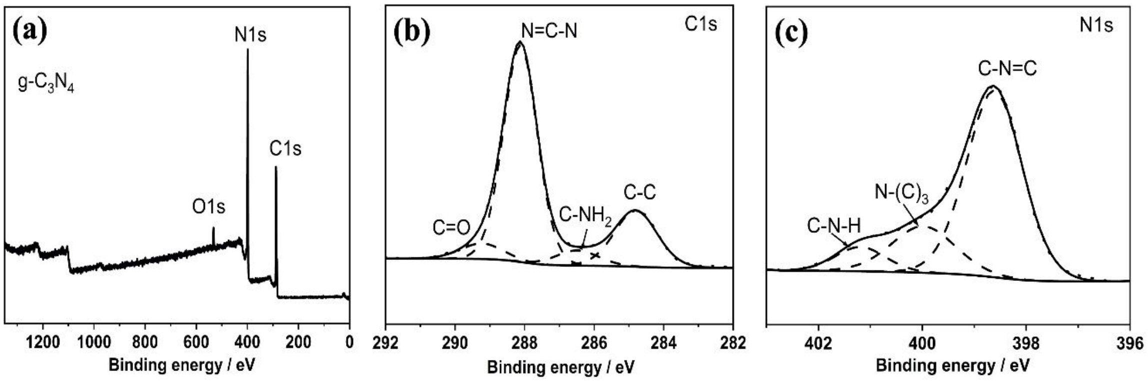

Figure S9. (a) XPS survey spectra of g-C_3N_4. (b) Higher resolution data for C1s of g-C_3N_4. (c) Higher resolution data for N1s of g-C_3N_4. Data are consistent with literature reports [3].

Table S2. XPS data analysis for g-C_3N_4.

Survey	Name	Peak BE	FWHM eV	Area (P) CPS.eV	Atomic %
	N1s	398.63	2.41	555643.04	47.91
	C1s	288.02	2.1	324040.72	47.46
	O1s	532.21	3.5	80819.36	4.63

C1s	Name	Peak BE	FWHM eV	Area (P) CPS.eV	Atomic %
	C1s N=C-N	288.12	1.15	5080.94	66.58
	C1s C-C	284.8	1.44	1651.98	21.61
	C1s C-NH2	286.49	1.44	444.62	5.82
	C1s C=O	289.23	1.44	456.46	5.98

N1s	Name	Peak BE	FWHM eV	Area (P) CPS.eV	Atomic %
	N1s N-$(C)_3$	399.99	1.44	2513.3	20.24
	N1s C-N=C	398.62	1.27	8874.25	71.41
	N1s C-N-H	401.2	1.14	1036.34	8.35

O1s	Name	Peak BE	FWHM eV	Area (P) CPS.eV	Atomic %
	O1s C=O	533.5	1.92	566.77	46.53
	O1s C-O	532	1.76	652	53.47

References

[1] Wang, L.; Carta, M.; Malpass-Evans, R.; McKeown, N. B.; Fletcher, P. J.; Lednitzky, D.; Marken, F., Hydrogen Peroxide versus Hydrogen Generation at Bipolar Pd/Au Nano-catalysts Grown into an Intrinsically Microporous Polyamine (PIM-EA-TB). *Electrocatal.* **2021**, *12* (6), 771–784.

[2] Landi, S.; Segundo, I.R.; Freitas, E.; Vasilevskiy, M.; Carneiro, J.; Tavares, C.J., Use and misuse of the Kubelka-Munk function to obtain the band gap energy from diffuse reflectance measurements. *Solid State Commun*. **2022**, *341*, 114573.

[3] Liu, G.; Niu, P.; Sun, C.H.; Smith, S.C.; Chen, Z.G.; Lu, G.Q.; Cheng, H.M., Unique Electronic Structure Induced High Photoreactivity of Sulfur-Doped Graphitic C_3N_4. *J. Amer. Chem. Soc*. **2010**, *132* (33), 11642–11648.
